# Deep sequencing of short capped RNAs reveals novel families of noncoding RNAs

**DOI:** 10.1101/gr.276647.122

**Published:** 2022-09

**Authors:** Michiel de Hoon, Alessandro Bonetti, Charles Plessy, Yoshinari Ando, Chung-Chau Hon, Yuri Ishizu, Masayoshi Itoh, Sachi Kato, Dongyan Lin, Sho Maekawa, Mitsuyoshi Murata, Hiromi Nishiyori, Jay W. Shin, Jens Stolte, Ana Maria Suzuki, Michihira Tagami, Hazuki Takahashi, Supat Thongjuea, Alistair R.R. Forrest, Yoshihide Hayashizaki, Juha Kere, Piero Carninci

**Affiliations:** 1 RIKEN Center for Integrative Medical Sciences, Yokohama, Kanagawa 230-0045, Japan;; 2 RIKEN Center for Life Science Technologies, Division of Genomic Technologies, Yokohama, Kanagawa 230-0045, Japan;; 3 RIKEN Preventive Medicine and Diagnosis Innovation Program, Wako, Saitama 351-0198, Japan;; 4 Integrated Program in Neuroscience, McGill University, Montreal, Quebec H3A 1A1, Canada;; 5 Mila, Montreal, Quebec H2S 3H1, Canada;; 6 RIKEN Omics Science Center (OSC), Yokohama, Kanagawa 230-0045, Japan;; 7 Genome Institute of Singapore, Agency for Science, Technology and Research (A*STAR), Singapore, 138632, Singapore;; 8 Harry Perkins Institute of Medical Research, QEII Medical Centre and Centre for Medical Research, The University of Western Australia, Nedlands, Perth, Western Australia 6009, Australia;; 9 Department of Biosciences and Nutrition, Karolinska Institutet, Huddinge 14157, Sweden;; 10 Stem Cells and Metabolism Research Program, University of Helsinki and Folkhälsan Research Center, Helsinki 00290, Finland;; 11 Human Technopole, Milan 20157, Italy

## Abstract

In eukaryotes, capped RNAs include long transcripts such as messenger RNAs and long noncoding RNAs, as well as shorter transcripts such as spliceosomal RNAs, small nucleolar RNAs, and enhancer RNAs. Long capped transcripts can be profiled using cap analysis gene expression (CAGE) sequencing and other methods. Here, we describe a sequencing library preparation protocol for short capped RNAs, apply it to a differentiation time course of the human cell line THP-1, and systematically compare the landscape of short capped RNAs to that of long capped RNAs. Transcription initiation peaks associated with genes in the sense direction have a strong preference to produce either long or short capped RNAs, with one out of six peaks detected in the short capped RNA libraries only. Gene-associated short capped RNAs have highly specific 3′ ends, typically overlapping splice sites. Enhancers also preferentially generate either short or long capped RNAs, with 10% of enhancers observed in the short capped RNA libraries only. Enhancers producing either short or long capped RNAs show enrichment for GWAS-associated disease SNPs. We conclude that deep sequencing of short capped RNAs reveals new families of noncoding RNAs and elucidates the diversity of transcripts generated at known and novel promoters and enhancers.

In eukaryotes, gene transcription by RNA polymerase (Pol) II generates a wide variety of messenger and long noncoding RNAs (mRNAs and lncRNAs, respectively). These transcripts are typically >1000 nt, and have a 7-methyl guanosine 5′ cap that distinguishes RNA Pol II products from transcripts generated by RNA Pols I and III. The cap analysis gene expression (CAGE) transcriptome profiling protocol (Takahashi et al. 2012) takes advantage of the 5′ cap to enrich for mRNAs and lncRNAs while avoiding the highly abundant ribosomal and transfer RNAs produced by RNA Pol I and III. Using CAGE and other deep sequencing approaches, the expression patterns and genomic extent of mRNAs and lncRNAs have been extensively studied and annotated by consortia such as ENCODE (Djebali et al. 2012) and FANTOM (The FANTOM Consortium and the RIKEN PMI and CLST (DGT) 2014). Almost all mRNAs have a poly(A) tail, with histone gene transcripts as a notable exception; both polyadenylated and nonpolyadenylated lncRNAs have been identified (Yang et al. 2011; Wu et al. 2017; Uszczynska-Ratajczak et al. 2018).

RNA Pol II also transcribes genes encoding spliceosomal and small nucleolar RNAs (Kiss 2004; Kufel and Grzechnik 2019). These transcripts are produced with a 7-methyl guanosine cap that is subsequently converted into a 2,2,7-trimethylguanosine cap (Mouaikel et al. 2002; Shuman 2007; Kufel and Grzechnik 2019), which is also recognized by CAGE. In addition, uncapped snoRNAs are produced by excision from introns of protein-coding genes (Kiss 2004). Mature spliceosomal and small nucleolar RNAs are nonpolyadenylated small RNAs, mostly with sizes up to 250 nt.

A third category of capped RNAs are produced at enhancers (Kim et al. 2010). These transcripts are relatively short, with sizes between 100 and 1000 nt, and usually do not have a poly(A) tail (Hou and Kraus 2021). Although enhancer RNAs have a 7-methyl guanosine 5′ cap and are therefore detected by CAGE (Andersson et al. 2014), their abundance is low owing to rapid degradation by the exosome complex (Schwalb et al. 2016; Hou and Kraus 2021).

High-throughput sequencing protocols for transcriptome profiling typically rely on reverse transcription to generate cDNA libraries from cellular RNA. Whereas an oligo(dT) primer that binds to the poly(A) tail can be used to reverse-transcribe polyadenylated RNA, current CAGE protocols make use of random primers to capture both polyadenylated and nonpolyadenylated RNA (The FANTOM Consortium and RIKEN Genome Exploration Research Group and Genome Science Group 2005; De Hoon and Hayashizaki 2008; Takahashi et al. 2012). As the efficiency of random primers is proportional to the size of the RNA and as the CAGE protocol includes purification steps to remove linker oligonucleotides while retaining longer products, short capped RNAs are largely absent from CAGE libraries. In particular, lowly expressed short enhancer RNAs and potentially other, currently unknown, short regulatory RNAs may be missed.

Here, we present a transcriptome profiling protocol specifically designed to capture short 5′ capped RNAs. We apply these protocols to RNA obtained in a 96-h time course of THP-1 monocytes, stimulated by phorbol 12-myristate 13-acetate (PMA) to induce their differentiation to macrophages. We compare the transcriptome of short capped RNAs to that of long capped RNAs, as observed in previously generated CAGE libraries for the same time course (Gažová et al. 2020), to uncover classes of short capped RNAs that are missing from current transcriptome data generated using existing protocols.

## Results

### A protocol for profiling short capped RNAs

**Figure 1.**
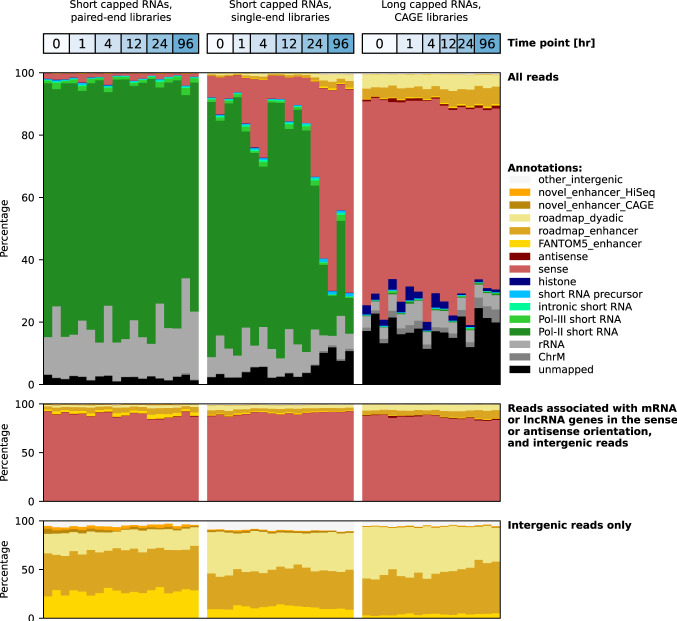
RNA composition per sequencing library. Pol II short RNAs include independently transcribed small nucleolar RNAs U3, U8, and U13; small Cajal body–specific RNAs 2 and 17; and spliceosomal RNAs except U6 and U6atac. Pol III short RNAs include transfer RNAs, spliceosomal RNAs U6 and U6atac, small ILF3/NF90-associated RNAs, the RNA component of the RNase P ribonucleoprotein, the RNA component of mitochondrial RNA processing endoribonuclease, the 7SK RNA component of the nuclear ribonucleoprotein, the 7SL RNA component of the signal recognition particle, Ro-associated RNAs, vault RNAs, and brain cytoplasmic RNA 1. Intronic RNAs include small nucleolar RNAs and small Cajal body–specific RNAs, except those transcribed by Pol II, and the *MALAT1*-associated small cytoplasmic RNA. Short RNA precursors include sequences that align within a 500-bp window upstream of and downstream from transfer RNAs, small nuclear RNAs, small nucleolar RNAs, and small Cajal body–specific RNAs but do not fully align to the mature RNA. The categories sense and antisense comprise transcripts associated with mRNAs and lncRNAs in the sense and antisense orientation, respectively.

THP-1 cells were stimulated by PMA to induce their differentiation from monocytes to macrophages. Sequencing libraries were produced as described in the Methods from RNA extracted at the same six time points during differentiation as in the previously published CAGE study (Gažová et al. 2020). Size selection was performed on the library products to select short capped RNAs (for details, see Methods) (Table 1). The product size distribution had two strong peaks corresponding to the sizes of spliceosomal RNA U1 and snoRNA U3 (defined in Supplemental Table S1), which are within the selected size range (Supplemental Fig. S1). Paired-end sequencing using a MiSeq sequencer (Supplemental Table S2) followed by functional categorization of the sequenced reads showed high enrichment for spliceosomal RNAs and small nucleolar RNAs (Fig. 1 [top]; Supplemental Fig. S2; Supplemental Table S3), showing that the protocol successfully captured short capped RNAs. In contrast, the CAGE data (Gažová et al. 2020) were predominantly associated with mRNA and lncRNA genes (Fig. 1). Consistent with the presence of a 5′ cap with a 5′-linked guanine, we found an enrichment of additional G nucleotides at the starting position of sequenced reads (Supplemental Fig. S3).

**Table 1.**
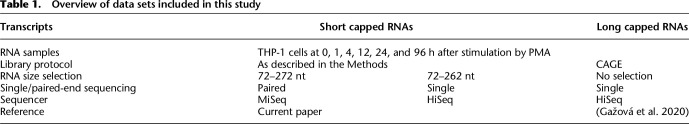
Overview of data sets included in this study

We repeated the library preparation protocol as technical replicates of the same RNA samples, followed by deep single-end sequencing from the 5′ side using the Illumina HiSeq 2000 sequencer (Supplemental Table S4). The library product size distribution showed the characteristic peaks for spliceosomal RNA U1 and snoRNA U3 (Supplemental Fig. S4); functional categorization of the sequenced reads confirmed that these libraries were dominated by short capped RNAs (Fig. 1 [top]). However, some samples at the later time points had markedly lower peaks for spliceosomal RNA U1 and snoRNA U3 (Supplemental Fig. S4A,B) and were instead highly enriched for reads aligning to the promoter region of the gene encoding diazepam binding inhibitor, acyl-CoA binding protein (*DBI*) (Supplemental Fig. S5A,B). We used Paraclu (Frith et al. 2008) on the combined paired-end and single-end libraries to define 151,555 transcription initiation peaks of short capped RNAs on the genome, created an expression table, and calculated the global dispersion for each sample as a measure of reproducibility (Supplemental Fig. S5C). As only a moderate increase in the dispersion value was observed at the later time points (Supplemental Fig. S5D), we concluded that the anomalous behavior of the expression at the *DBI* promoter had only minor effects on the overall reproducibility of these samples.

In this and all subsequent analyses, all reads mapping to known classes of short RNAs were excluded (see Methods) (Fig. 1 [middle]).

### Comparative analysis of gene-associated short and long capped RNAs

**Figure 2.**
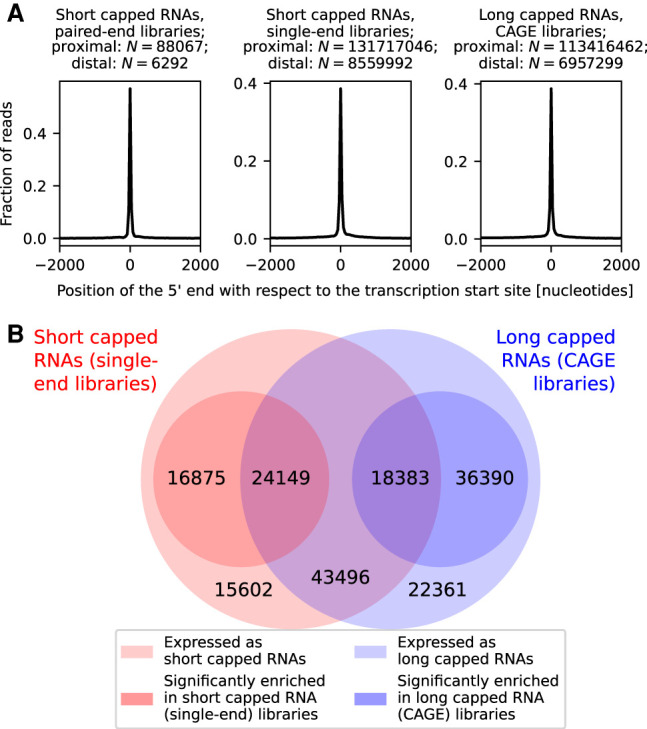
Gene-associated short and long capped RNAs. (*A*) Position of the 5′ end of short and long capped RNAs, associated in the sense orientation with annotated genes, relative to the transcription start site of the gene. (*B*) Venn diagram of transcription initiation peaks associated with genes in the sense orientation. The *outer* circles represent peaks expressing short capped RNAs (red *outer* circle) and long capped RNAs (blue *outer* circle). The *inner* circles represent peaks with a significantly higher expression of short capped RNAs than long capped RNAs (red *inner* circle) or a significantly higher expression of long capped RNAs than short capped RNAs (blue *inner* circle).

We applied Paraclu (Frith et al. 2008) to the combined short and long capped RNA data (single-end libraries and CAGE libraries, respectively), generating a single set of 286,343 transcription initiation peaks, and created an expression table with the read counts of short and long capped RNAs for each peak. In total, 177,256 transcription initiation peaks were associated in the sense orientation with transcribed mRNA and lncRNA genes, with a transcription start site located in the CAGE-defined promoter of the associated gene (Fig. 2A). About half (86,028) of those peaks generated both short and long capped RNAs (Fig. 2B). Short and long capped RNA expression of these peaks was highly correlated with each other across peaks (Pearson's correlation = 0.68, *P* < 10^−100^) (Supplemental Fig. S6) and was proportional to each other for each peak during the time course (Pearson's correlation = 0.31, *P* < 10^−100^) (Supplemental Fig. S7), suggesting that short and long capped RNA expression is coregulated. However, 32,477 peaks produced only short capped RNAs and 58,751 peaks only long capped RNAs (Fig. 2B), suggesting that different peaks have different expression ratios of short capped RNAs to long capped RNAs. Peaks with a statistically significant deviation in the relative expression of short and long capped RNAs were identified using DESeq2 (requiring an adjusted *P* < 0.05) (Love et al. 2014) and are henceforth referred to as peaks enriched for short or long capped RNAs (Supplemental Fig. S6); we note that short and long capped RNA expression levels cannot be compared directly to each other owing to the difference in RNA composition of short and long capped RNA libraries (Fig. 1 [top]). More than half of the 177,256 gene-associated peaks were enriched for short capped RNAs (41,024 peaks) or long capped RNAs (54,773 peaks) (Fig. 2B).

**Figure 3.**
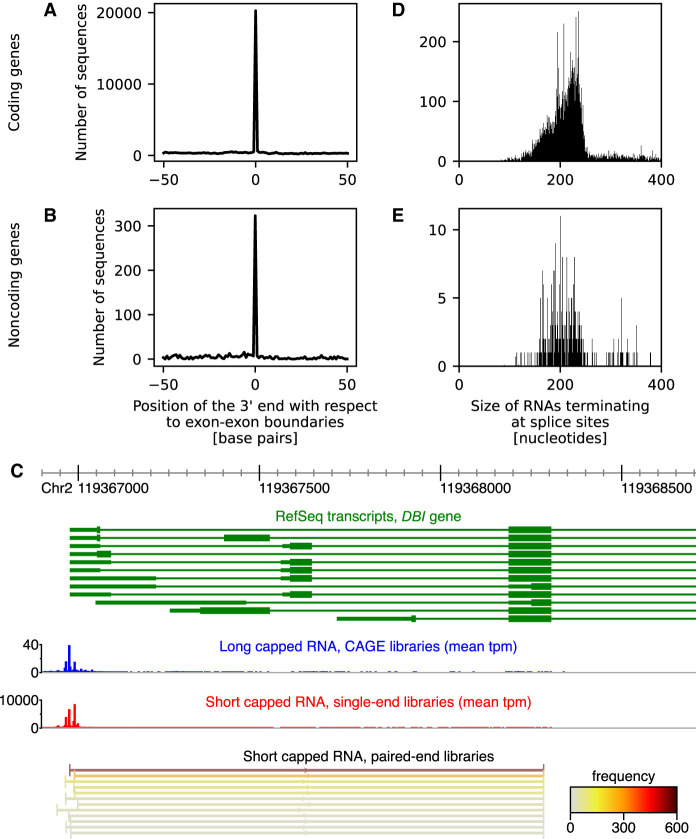
Position of the 3′ end of short capped RNAs aligning to coding (*A*) or noncoding (*B*) genes, relative to their splice sites. (*C*) 5′ Region of the diazepam binding inhibitor, acyl-CoA binding protein (*DBI*) gene. RefSeq transcripts associated with the *DBI* gene are shown in green. The position of the 5′ end of long capped RNAs is shown in blue as an expression histogram of the mean number of tags per million (tpm) observed in the CAGE libraries; the position of the 5′ end of short capped RNAs is shown in red as an expression histogram of the mean number of tpm observed in the single-end libraries. The 5′ and 3′ end of short capped RNAs aligning to the mature mRNA, colored by total read frequency, as observed in the paired-end libraries at the *bottom* (only the top expressed RNAs are shown), revealing that the short capped RNAs terminated at one specific position within an exon of *DBI*. (*D*) Size of short capped RNAs terminating at splice sites of coding genes. (*E*) Size of short capped RNAs terminating at splice sites of noncoding genes.

Using the paired-end libraries to determine both the 5′ and the 3′ end of transcripts, we found that gene-associated short capped RNAs are typically shorter than the corresponding mRNA or lncRNA transcript (Supplemental Fig. S8) and may represent truncated transcripts. Such truncated transcripts were found at 7170 coding and 768 noncoding genes. The 3′ end of the short capped RNA was significantly enriched at splice site boundaries (*P* < 10^−100^, Fisher's combined probability of binomial tests), with 2930 (41.5%) out of 7068 coding genes with spliced mRNA transcripts and 141 (21.1%) out of 668 noncoding genes with spliced lncRNA transcripts having significantly (*P* < 0.05, binomial test) enriched termination at splice site boundaries (Fig. 3A,B). On average, 55.6% and 73.6% of short capped RNAs aligning to these coding and noncoding genes terminated at splice sites. For *DBI*, however, the preferred truncation site was located inside an exon (Fig. 3C).

Short capped RNAs terminating at splice sites had a median size of 216 nt (Fig. 3D,E). In contrast, Start-Seq (Nechaev et al. 2010) in THP-1 cells stimulated by PMA (Heinz et al. 2018) revealed transcripts with sizes 25–65 nt associated with Pol II pausing. Although the expression of short capped RNAs was correlated with the Start-Seq tag count (Pearson's correlation = 0.44, *P* < 10^−100^) (Supplemental Fig. S9A), 72,712 and 1519 peaks, respectively, were expressed in the short capped RNA single-end libraries and the Start-Seq libraries only, and 4905 and 8015 peaks had a statistically significantly higher relative expression in the short capped RNA libraries and Start-Seq libraries, respectively (Supplemental Fig. S9B).

### Enhancer expression of short and long capped RNAs

We identified enhancer regions by their bidirectional transcription (Andersson et al. 2014) of short or long capped RNAs, yielding 11,307 and 18,167 enhancers, respectively. The majority of these, 7644 (67.6%) and 10,455 (57.5%), respectively, were confirmed by overlapping enhancer regions annotated by the Roadmap Epigenomics Project (Roadmap Epigenomics Consortium et al. 2015). In contrast, only 3881 (34.3%) and 3930 (21.6%) enhancers, respectively, overlapped the 63,285 enhancers previously discovered from 1829 CAGE libraries from FANTOM5 (Rennie et al. 2018). By down-sampling the short and long capped RNAs, we found that enhancer prediction was far from saturation (Supplemental Fig. S10), suggesting that many enhancers remain to be discovered. Additionally, 7779 (68.8%) and 14,663 (80.7%) of the enhancers were predicted only from the short and long capped RNA data, respectively, suggesting that sequencing of short and long capped transcripts reveals overlapping but distinct sets of enhancers.

**Figure 4.**
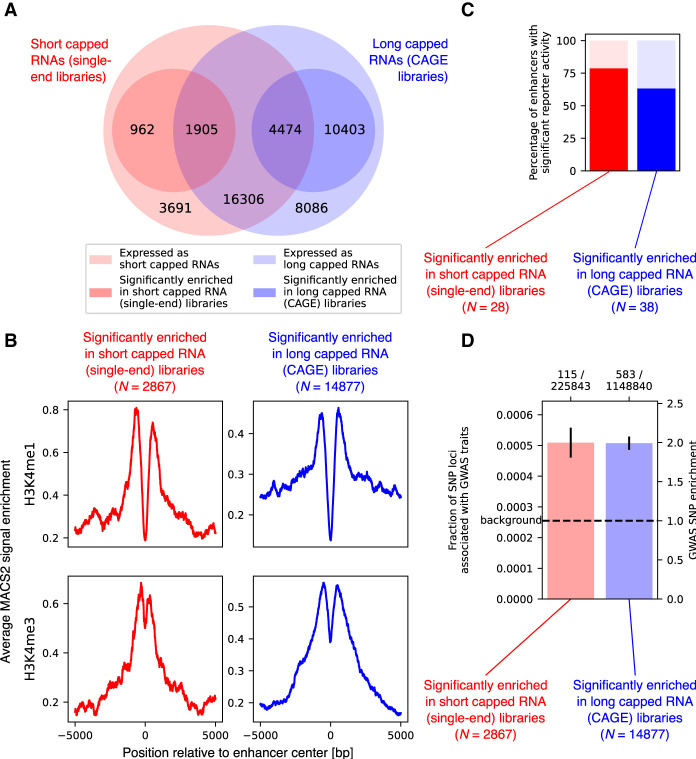
Enhancer expression of short and long capped RNAs. (*A*) Venn diagram of predicted enhancers. The *outer* circles represent predicted enhancers expressing short capped RNAs (red *outer* circle) and long capped RNAs (blue *outer* circle). The *inner* circles represent predicted enhancers significantly enriched for short capped RNAs expression (red *inner* circle) or for long capped RNA expression (blue *inner* circle). (*B*) Reporter activity of predicted enhancers with enriched for expression of short or long capped RNAs. (*C*) H3K4me1 and H3K4me3 epigenetic marker signal for enhancers and active promoters, respectively, at promoters enriched for short or long capped RNAs. (*D*) Fraction of SNP loci associated with GWAS traits overlapping predicted enhancers enriched for short or long capped RNA expression; error bars indicate the standard deviation of the fraction. The number of GWAS-associated SNP loci and the total number of SNP loci are shown as a ratio *above* the graph. The dashed line shows the genome-wide fraction of SNP loci associated with GWAS traits as the background level.

We merged the enhancers predicted from the short and long capped RNA data with the FANTOM5 enhancers into a joint set of 45,827 expressed enhancers and analyzed their expression as short and long capped RNAs. Of the joint set of enhancers, 22,685 (49.5%) were expressed both as short and as long capped RNAs, 4653 (10.2%) were observed in the short capped RNA data only, and 18,489 (40.3%) were observed in the long capped RNA data only (Fig. 4A). Expression levels of short and long capped RNAs were correlated across enhancers (Pearson's correlation 0.44, *P* < 10^−100^) (Supplemental Fig. S11). Using differential expression analysis to compare the expression ratio of short and long capped RNAs across enhancers, we found 2867 enhancers significantly (DESeq2 adjusted *P* < 0.05) enriched for short capped RNAs and 14,877 enhancers significantly enriched for long capped RNAs (Fig. 4A; Supplemental Fig. S11). Of these, 962 and 10,403 enhancers only expressed either short or long capped RNAs, respectively (Fig. 4A).

Predicted enhancers preferentially expressing short or long capped RNAs were both enriched for the H3K4me1 epigenetic marker for enhancers and the H3K4me3 epigenetic marker for active promoters (data obtained from Shi et al. 2021a) compared with randomly selected regions (all with *P* < 10^−100^, Mann–Whitney *U* test) (Fig. 4B), with the H3K4me1/H3K4me3 ratio significantly greater for enhancers enriched for short capped RNAs compared with enhancers enriched for long capped RNAs (*P* = 0.0012, *Z*-test). Using previously published reporter assay data (Andersson et al. 2014), we found that 79% and 63% of predicted enhancers preferentially expressing short or long capped RNAs, respectively, acted as enhancers (Fig. 4C); the difference was not statistically significant (*P* = 0.28, Fisher's exact test). Single-nucleotide polymorphism (SNP) loci were enriched for traits in genome-wide association studies (GWAS) (Farh et al. 2015; Demenais et al. 2018) by a factor of 2.0 compared with background both in enhancers enriched for short capped RNAs and in enhancers enriched for long capped RNAs (*P* = 1.7 × 10^−11^ and *P* = 1.3 × 10^−50^, respectively, binomial test) (Fig. 4D); the difference was not statistically significant (*P* = 0.96, Fisher's exact test).

## Discussion

By sequencing short capped RNAs, we were able to capture transcripts that are excluded from existing transcriptome sequencing approaches that target longer transcripts such as mRNAs and lncRNAs. In contrast, our sequencing libraries consisted of well-known short capped transcripts such as small nucleolar and spliceosomal RNAs, as well as novel families of short capped RNAs. As the library preparation protocol only captures RNAs with a 3′ hydroxyl group, additional classes of short capped RNAs may be discovered by introducing enzymes to convert any 3′ phosphate or cyclic phosphate group before ligation (Shi et al. 2021b). As an alternative to our protocol, short capped RNAs can be selected during library preparation by enzymatic removal of the 5′ cap followed by gel purification (Gu et al. 2012).

The RNA size selection limits of 72–272 nt were chosen to enrich for short enhancer RNAs likely to be missed by CAGE, which is inefficient for transcripts shorter than ∼250 nt. RNAs even shorter than our selected size range, which may also be functionally active, are captured by Start-Seq (Nechaev et al. 2010). Selecting RNAs with sizes between those captured by Start-Seq and those captured by CAGE allowed us to maximize the chance of finding RNAs not yet described in the scientific literature.

Both at mRNA and lncRNA genes, we found short capped RNAs that overlap previously annotated long transcript isoforms in the sense orientation. As the short transcripts had very specific 3′ ends at each gene, they are unlikely to be degradation products of the longer transcript but may be owing to RNA polymerase pausing before continuing to generate the full-length transcript. Alternatively, premature termination of transcription may act as a mechanism for gene expression regulation, as has been observed in yeast (Porrua and Libri 2015). As the 3′ end of the short capped transcripts tended to coincide with splice sites, they may also be formed as part of a quality-control step during splicing or as a byproduct of the formation of circular RNA.

In both the long and the short capped RNA data set, we identified a large number of candidate novel enhancers that were not observed in the FANTOM5 CAGE data, comprising a large number of different primary cell types, cell lines, and tissues (Rennie et al. 2018). This indicates that deeper sequencing of transcription initiation events in a single cell type can reveal many enhancers that are missed in broad expression atlases. We found that some enhancers preferentially generate long transcripts, whereas others preferentially generate short enhancer RNAs, which may be missed by profiling methods that capture long transcripts.

Most of the short capped RNA libraries consist of known short RNAs of snoRNAs and small Cajal body–associated RNAs that have a 2,2,7-trimethylated cap. In contrast, the novel gene- and enhancer-associated short capped RNAs described in this work are likely to have a usual 7-monomethyl cap (Supplemental Fig. S3). This suggests that the protocol can be improved by using antibodies against 2,2,7-trimethylated caps to deplete such highly expressed known short RNAs from the library, allowing deeper sequencing of novel short capped RNAs. Additionally, the variability between libraries prepared from the same RNA, likely caused by nonuniform amplification by PCR, suggests that the quantitativeness of the protocol may be improved by using unique molecular identifiers (UMIs) to remove PCR duplicates.

## Methods

### THP-1 differentiation time course

Time course samples were prepared by seeding 2 million THP-1 cells/well in 10-cm plates with media containing penicillin/streptomycin and 30 ng/mL PMA. RNA was extracted at 0, 1, 4, 12, 24, and 96 h of stimulation by PMA with three replicates.

### Library preparation for paired-end sequencing of short capped RNAs

Using 1 μg of RNA from each of the 18 samples (Supplemental Table S2), the 3′ adaptor (5′-rApp-CTGTAGGCACCATCAAT-ddC-3′, with r, d, and p representing RNA, DNA, and phosphate) was ligated to the 3′ end of total RNAs with 200 U of T4 RNA ligase 2, truncated (NEB) in 1 × buffer, 8 mM MgCl_2_, and 20 U of RNaseOUT (Thermo Fisher Scientific) for 60 min at 20°C. After purification of RNAs with a RNeasy MinElute kit (Qiagen), the RNAs were reverse-transcribed with 200 U of SuperScript III, 1 µL of 10 mM dNTPs, 4 µL of 5 × reaction buffer, 1 µL of 0.1 M DTT, 1 µL of RNaseOUT, and 2.5 µM of primer (5′-CCGATCTCACCTCCTGCATCATTGATGGTGCCTACAG-3′) for 60 min at 50°C. After the RT reaction, 20 µL of RNA–cDNA hybrids was purified with 36 µL of AMPure RNA clean XP. Next, the 5′ cap guanine of the RNA–cDNA hybrids was trapped with the cap-trapping method, which has been described previously (Murata et al. 2014). Briefly, the diol group of the 5′ cap guanine of RNA–cDNA hybrids were oxidized by NaIO_4_; noncapped hybrids were digested with RNase ONE; and the 5′ capped hybrids were biotinylated and trapped by MPG streptavidin beads. After releasing the single-stranded cDNAs from RNAs, the 5′ linker (5′nAnT-iCAGE_01 N6: 5′-CGACGCTCTTCCGATCTXXXNNNNNN-Phos-3′; 5′nAnT-iCAGE_01 GN5: 5′-CGACGCTCTTCCGATCTXXXGNNNNN-Phos-3′; 5′nAnT-iCAGE_01 Dwn: 5′-Phos YYYAGATCGGAAGAGCGTCG Phos-3′; where the 5′ barcode XXX is listed in Supplemental Table S2 and YYY is the reverse complement of XXX) was ligated to the 3′ end of the single-stranded (ss)cDNAs (Murata et al. 2014). Fifteen microliters of 5′ linker ligated sscDNAs was then purified with 27 µL of AMPure XP and amplified with 0.25 U of TaKaRa ex Taq hot start and primers (5′-CAAGCAGAAGACGGCATACGAGATXXXXXXCCGATCTCACCTCCTGCATCATTGATGGT-3′, where XXXXXX represents the 3′ index [Supplemental Table S2], and 5′-AATGATACGGCGACCACCGAGATCTACACTCTTTCCCTACACGACGCTCTTCCGATCT-3′) with 10 cycles of (1 min at 95°C, 15 sec at 95°C, 10 sec at 55°C, 2 min at 68°C) and 10 cycles of (15 sec at 95°C, 10 sec at 65°C, 2 min at 68°C, keep at 4°C). The amplified sample was purified by AMPure XP purification, and 5 ng of each sample, or all if <5 ng was available, was pooled in one library. Pooled libraries were then concentrated to 30 µL with miVAC DNA (Genevac, SP Scientific DNA-10000-G00). Products with sizes of 200–400 bp (corresponding to RNA sizes of 72–272 nt) were selected using the Pippin Prep instrument (Sage Science). Forty-two microliters of size-selected libraries was purified with 75.6 µL of AMPure XP purification. The size distribution of library products before and after size selection were measured by a Bioanalyzer (DNA high-sensitivity kit, Agilent) (Supplemental Figs. S1, S2); 8 pM of libraries were sequenced with Illumina MiSeq 75 cycles kit (PE read1 is 36 cycles, read2 is 33 cycles, and index read is six cycles). The pooled library also contained 18 samples of transfection experiments in which specific short capped RNAs were knocked down using LNA-modified GapmeR antisense oligonucleotides (Supplemental Table S2). However, the corresponding data were removed from the analysis as we were not able to confirm a successful knockdown.

### Library preparation for single-end sequencing of short capped RNAs

This library was prepared using all remaining (0.6–9.1 μg) total RNA from each of the 18 time course samples (Supplemental Table S4) using the same library preparation protocol as for the paired-end sequencing of short capped RNAs, except that 14 PCR cycles were performed for library amplification. The size distribution of library products before size selection was measured by a Bioanalyzer (Supplemental Fig. S4). An equal molarity of cDNAs from each of the 18 libraries were combined into a single pooled library, followed by size selection of library products with a size of 200–390 bp (corresponding to RNA sizes of 72–262 nt). The library amount was measured by PicoGreen. The size distribution of library products of the pooled library after size selection was evaluated with Bioanalyzer (DNA high sensitivity kit, Agilent) (Supplemental Fig. S4C). The libraries were sequenced using the Illumina HiSeq 2000 SR sequencing (50 cycles) kit.

### RNA transcript models

We downloaded the NCBI RefSeq annotations on December 10, 2020, and extracted 37 ribosomal RNA sequences, 36 small nuclear RNAs, six small cytoplasmic RNAs, 541 small nucleolar RNAs, four Ro-associated RNAs, 136 histone mRNA transcripts, the RNA component of mitochondrial RNA processing endoribonuclease (*RMRP*), 29 small Cajal body–specific RNAs, the RNA component of the RNase P ribonucleoprotein (*RPPH1*), 28 small ILF3/NF90-associated RNAs, telomerase RNA component (*TERC*), four vault RNAs, three transcript isoforms of metastatis associated lung adenocarcinoma transcript 1 (*MALAT1*), 124 small nucleolar RNA host gene transcripts, 115,876 mRNA transcripts (excluding histone mRNAs), and 45,299 lncRNA transcripts. The small nuclear RNAs and small nucleolar RNAs were supplemented by annotations from Ensembl release 100 (Zerbino et al. 2018) to create a set of 2073 small nuclear RNA sequences and a set of 1034 small nucleolar RNA sequences. Category definitions of these noncoding transcripts are provided in Supplemental Table S1. RepeatMasker annotations (downloaded from UCSC on October 21, 2016), tRNA genes identified by tRNAscan-SE (downloaded from UCSC on April 26, 2020) (Chan and Lowe 2019), and tRNA annotations in the Entrez Gene database (downloaded from NCBI on April 26, 2020) (Brown et al. 2015) were combined into a set of 2089 mature tRNA sequences and their genomic locations. GENCODE transcripts were obtained from GENCODE release 34. FANTOM-CAT (Hon et al. 2017) transcript models for human genome hg38 were obtained from FANTOM6 (Ramilowski et al. 2020).

Splice boundaries were obtained from the 115,397 RefSeq mRNA and 43,674 RefSeq lncRNA transcript models that were mapped to the human genome assembly hg38.

### Tag extraction and mapping

TagDust version 2.13 (Lassmann 2015) was used to extract the RNA sequences from the paired-end and single-end data of short capped RNAs.

CAGE data were downloaded as raw sequences from the NCBI Sequence Read Archive (SRA; https://www.ncbi.nlm.nih.gov/sra) under accession numbers ERR4221456, ERR4221458, ERR4221472, ERR4221496, ERR4221459, ERR4221476, ERR4221452, ERR4221508, ERR4221468, ERR4221485, ERR4221457, ERR4221481, ERR4221506, ERR4221454, ERR4221469, and ERR4221470. Following the description of Gažová et al. (2020), CAGE tags were extracted using cutadapt version 1.18 using the command “cutadapt -g XnnnCAGCAG…TCGTATGCCGTCTTCTGCTTG ‐‐match-read-wildcards ‐‐discard-untrimmed ‐‐minimum-length 20 ‐‐overlap 6,” where nnn is the linker sequence for each sample.

Start-Seq data (Heinz et al. 2018) were downloaded from NCBI SRA (Katz et al. 2022) under accession numbers SRR7071452 and SRR7071453.

From this point onward, the paired-end and single-end short capped RNA data, the CAGE data of long capped RNAs, and the Start-Seq data were processed using the same pipeline.

To identify sequences derived from known classes of short capped RNAs as much as possible and exclude them from the further analysis, we performed Needleman–Wunsch global alignment using Biopython version 1.79 (Cock et al. 2009) for each sequence as the query against the mitochondrial genome, ribosomal RNA, transfer RNAs, small nuclear RNAs, small cytoplasmic RNAs, small nucleolar RNAs, Ro-associated RNAs, histone mRNAs, *RMRP*, small Cajal body–specific RNAs, *RPPH1*, small ILF3/NF90-associated RNAs, *TERC*, vault RNAs, *MALAT1* transcript isoforms, and small nucleolar RNA host gene transcripts successively as target sets, with a match score of +1, a mismatch score of −1, and a gap score of −1, except for gaps before or after the query sequence, for which the gap score was zero. Alignments with an alignment score less than 0.8× the length of the query sequence were discarded. Alignments with the highest score were retained. If multiple such alignments were found, alignments against the shortest target transcript were retained; of those, the alignments with the shortest extent along the target sequence were retained. Sequences that aligned successfully to a target set were excluded for subsequent target sets. We used BWA (Li and Durbin 2009) with arguments “mem -O 0 -E 1 -A 1 -B 1 -T 10 -k 10 -c 100000000 -a -Y” to align sequences to the mRNA, lncRNA, GENCODE, and FANTOM-CAT transcript sets, as well as to the genome, requiring a minimum alignment score of 0.9 × the length of the query sequence.

### Functional categorization

Sequences aligning to the mitochondrial genome, to ribosomal RNA, or to histone gene transcripts were categorized as ChrM, rRNA, and histone, respectively. Sequences aligning to transfer RNAs; small cytoplasmic RNAs brain cytoplasmic RNA 1 (*BCYRN1*) and the 7SL RNA component of signal recognition particle; small nuclear RNAs U6, U6atac, and 7SK; Ro-associated RNAs; *RMRP*; *RPPH1*; small ILF3/NF90-associated RNAs; or vault RNAs were categorized as Pol III short RNAs. Sequences aligning to small nuclear RNAs U1, U2, U4, U4atac, U5, U7, U11, or U12, to small nucleolar RNAs U3, U8, or U13, or to small Cajal body–specific RNAs 2 or 17 were categorized as Pol II short RNAs. Sequences aligning to other small nucleolar RNAs, to other small Cajal body–specific RNAs, or to *MALAT1*-associated small cytoplasmic RNA (*mascRNA*) were categorized as intronic short RNAs. Remaining sequences were categorized based on their overlap with the following genomic regions:

- Short RNA precursor regions, defined as genomic regions within 500 bp of a small nuclear RNA gene, a transfer RNA gene, a small nucleolar RNA gene, or a small Cajal body–specific RNA gene;
- FANTOM5 enhancer regions;
- Roadmap Epigenomics enhancer regions;
- Roadmap Epigenomics dyadic regions;
- Novel enhancer regions predicted from the short capped RNA data (single-end libraries);
- Novel enhancer regions predicted from the long capped RNA data (CAGE libraries);
- FANTOM-CAT genes overlapping in the sense orientation; and
- FANTOM-CAT gene overlapping in the antisense orientation.

For each sequence, the categories were evaluated in the order listed and were assigned to the sequence based on the first overlap found. Any remaining sequences were categorized as “other_intergenic” (Fig. 1 [bottom]).

For the purpose of defining transcription initiation peaks, quantifying their expression levels, and quantifying the expression levels of enhancers, we excluded all multimapping reads, as well as reads aligning to the mitochondrial chromosome, ribosomal RNAs, transfer RNAs or their precursors, small nucleolar RNAs or their precursors, small Cajal body–specific RNAs or their precursors, small nuclear RNAs or their precursors, small cytoplasmic RNAs, Ro-associated RNAs, vault RNAs, small ILF3/NF90-associated RNAs, or histone transcripts.

### Enhancer annotations

FANTOM5 enhancers (Andersson et al. 2014) for human genome assembly hg38 were obtained from Rennie et al. (2018). Roadmap Epigenomics promoters, enhancers, and dyadic regions (Roadmap Epigenomics Consortium et al. 2015) were downloaded for human genome assembly hg19 from https://egg2.wustl.edu/roadmap/data/byDataType/dnase/, subdirectories BED_files_prom, BED_files_enh, and BED_files_dyadic, respectively. Each set was merged over the 111 Roadmap reference epigenomes and then lifted over to human genome assembly hg38 using liftOver (Hinrichs et al. 2006).

### Analysis of transcripts terminating at splice sites

Using the paired-end sequencing data, we calculated the background size distribution of all short capped RNAs aligning to mRNA or lncRNA transcripts. Full-length alignments, defined as those that extended over >90% of the mRNA or lncRNA transcript, were excluded. For each mRNA or lncRNA transcript, we counted the number of short capped RNAs aligning to the transcript that terminated at one of its splice sites. Next, we anchored the 5′ end of each short capped RNA and calculated the probability of the short capped RNA to terminate at a splice boundary if its size was chosen randomly from the background size distribution. We averaged these probabilities over all short capped RNAs aligning to transcripts associated with each gene to find the background probability of a short capped RNA to terminate at a splice boundary. The statistical significance was calculated for each gene by applying the binomial test to the number of short capped RNAs aligning to transcripts associated with the gene, the number of short capped RNAs terminating at splice sites, and the background probability calculated for the gene.

### Comparison of short and long capped RNA expression

Transcription initiation peaks were generated by running Paraclu (Frith et al. 2008) on the combined long capped RNA (CAGE) and short capped RNA (single-end) data sets, requiring at least 10 tags for each peak. Differential expression analysis was performed using DESeq2 version 1.30.1 (Love et al. 2014), requiring an adjusted *P*-value <0.05; global dispersion values were estimated using glmGamPoi (Ahlmann-Eltze and Huber 2021).

To calculate expression log_2_ fold changes, we first calculated the dispersion in the long and short capped RNA data separately and took their mean. Next, we used DESeq2 to estimate the library size factors, divided them by their median, applied the variance stabilizing transformation $x\to \left(2\;\mathrm{arcsinh}(\sqrt{ax})-\log(a)-\log(4)\right)/\log(2)$ (Anders and Huber 2010), calculated the mean for each time point in the long and short capped RNA data separately, and subtracted their average across time points. Peaks were excluded from Supplemental Figure S7 if neither the long capped RNAs nor the short capped RNAs were differentially expressed during the time course as assessed by one-way ANOVA, requiring *P* < 0.05 for significance.

### Prediction of novel enhancers

Enhancers were predicted as described previously (Andersson et al. 2014). We extracted all live transcript isoforms from the Entrez Gene database (downloaded from NCBI on April 26, 2020) (Brown et al. 2015) with gene type “protein-coding,” “pseudo,” “ncRNA,” “snRNA,” “scRNA,” “snoRNA,” “other,” and “unknown.” All exonic regions of these transcripts as well were excluded from enhancer prediction. All genomic regions within 500 bp upstream of or downstream from the TSS of transcripts other than “snRNA,” “scRNA,” and “snoRNA” were also excluded. All sequencing data aligning to the mitochondrial genome, ribosomal RNA, transfer RNAs or their precursors, small nucleolar RNAs or their precursors, small Cajal body–specific RNAs or their precursors, small nuclear RNAs or their precursors, small cytoplasmic RNAs, Ro-associated RNAs, vault RNAs, small ILF3/NF90-associated RNAs, and histone mRNAs were excluded, as well as sequencing data with alignments to spliced transcripts that overlap exon–exon boundaries and sequencing data mapping to more than one genomic location.

### Analysis of H3K4me1 and H3K4me3 epigenetic markers

Previously published (Shi et al. 2021a) ChIP-seq data for the H3K4me1 and H3K4me3 epigenetic marker were obtained from the NCBI Gene Expression Omnibus (GEO; https://www.ncbi.nlm.nih.gov/geo/) (Barrett et al. 2013) under accession numbers GSM5379664 and GSM5379671, respectively. For each enhancer, the mean ChIP-seq score was calculated in a window of ±5000 bp with respect to the center of the enhancer. Using linear regression separately for enhancers significantly enriched in short capped RNAs and for enhancers significantly enriched in long capped RNAs, we estimated the slope of the regression line between the H3K4me1 and H3K4me3 scores across enhancers, as well as its standard deviation. This slope represents the constant of proportionality between the H3K4me1 score and the H3K4me3 score. The *Z*-score was calculated as the difference in the constant of proportionality for enhancers enriched in short capped RNAs and for enhancers enriched in long capped RNAs, divided by the root mean square of the two standard deviations.

### GWAS SNP enrichment analysis

Genomic positions of SNPs were downloaded from the NCBI dbSNP database (https://www.ncbi.nlm.nih.gov/snp/) build 154 of May 1, 2020 (Sherry et al. 2001). The GWAS catalog of genome-wide association studies (Buniello et al. 2019) released on April 7, 2022, was downloaded from EMBL-EBI on May 20, 2022.

## Data access

The paired-end and single-end short capped RNA sequencing data generated in this study have been submitted to the DNA Data Bank of Japan (DDBJ) under accession number DRA013398 (https://ddbj.nig.ac.jp/resource/sra-submission/DRA013398). All custom scripts created in this study are available as Supplemental Code.

## Supplementary Material

Supplemental Material
