# Supplementary material for: Deep sequencing of short capped RNAs reveals novel families of noncoding RNAs
Source: Genome Res. 2022 Sep;32(9):1727–35. doi: 10.1101/gr.276647.122 (PMC9528987; doi:10.1101/gr.276647.122)
Supplement: Supplemental Material [file supp_32_9_1727__DC1.html]

Deep sequencing of short capped RNAs reveals novel families of noncoding RNAs — Supplemental Material 

# Deep sequencing of short capped RNAs reveals novel families of noncoding RNAs

## Supplemental Material

- Supplemental\_Fig\_S1.pdf
- Supplemental\_Fig\_S2.pdf
- Supplemental\_Fig\_S3.pdf
- Supplemental\_Fig\_S4.pdf
- Supplemental\_Fig\_S5.pdf
- Supplemental\_Fig\_S6.pdf
- Supplemental\_Fig\_S7.pdf
- Supplemental\_Fig\_S8.pdf
- Supplemental\_Fig\_S9.pdf
- Supplemental\_Fig\_S10.pdf
- Supplemental\_Fig\_S11.pdf
- Supplemental\_Table\_S1.xlsx
- Supplemental\_Table\_S2.xlsx
- Supplemental\_Table\_S3.xlsx
- Supplemental\_Table\_S4.xlsx
- Supplemental\_Code.zip
