# Supplementary material for: Deep sequencing of short capped RNAs reveals novel families of noncoding RNAs
Source: Genome Res. 2022 Sep;32(9):1727–35. doi: 10.1101/gr.276647.122 (PMC9528987; doi:10.1101/gr.276647.122)
Supplement: Supplemental Material [file supp_gr.276647.122_Supplemental_Fig_S1.pdf]

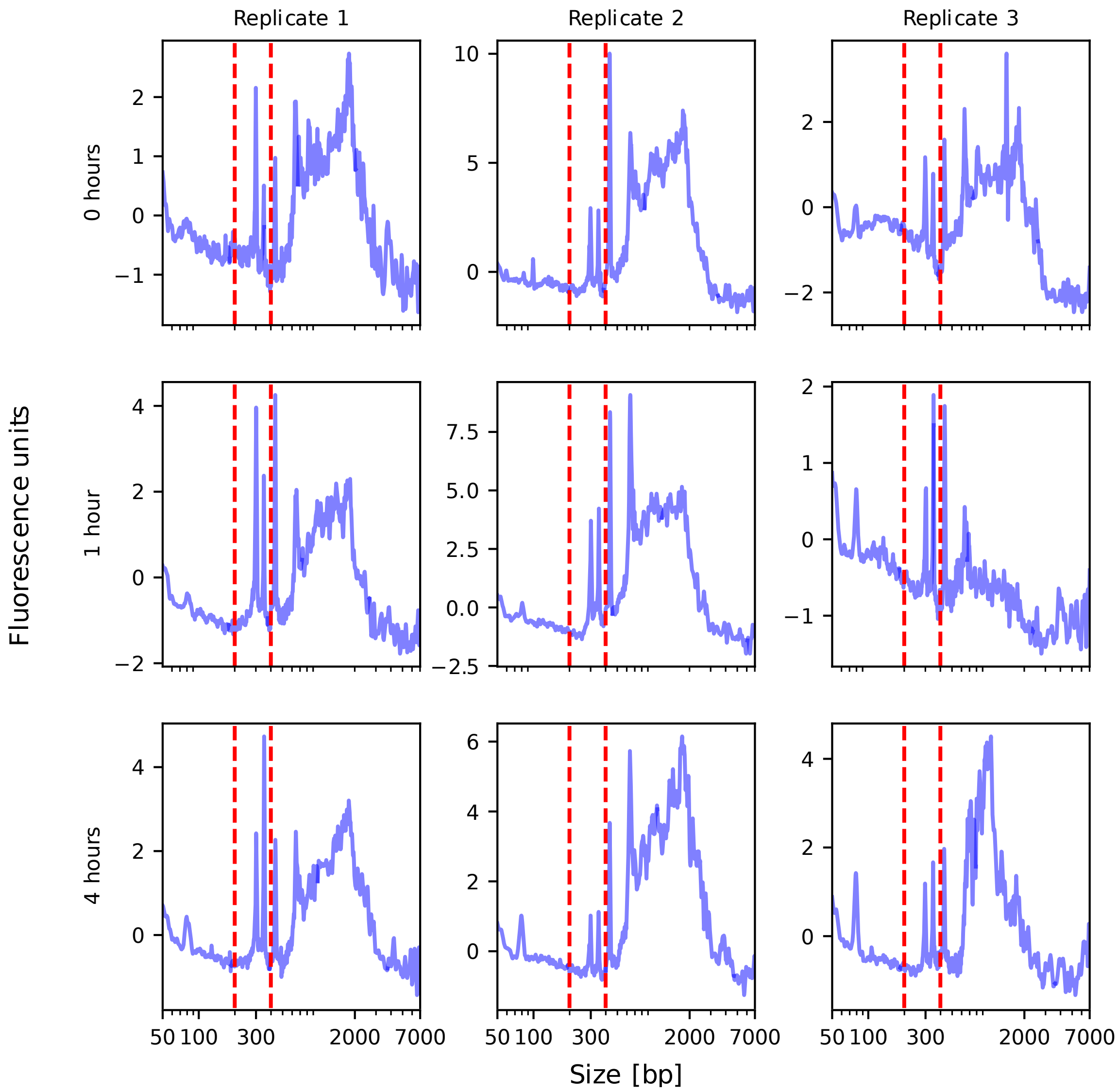

Supplementary Figure S1A. Product sizes of selected paired-end libraries of short capped RNAs before size selection were measured by Bioanalyzer. The product size distribution is shown for the full size range. The selected size limits are indicated by red dashed lines.

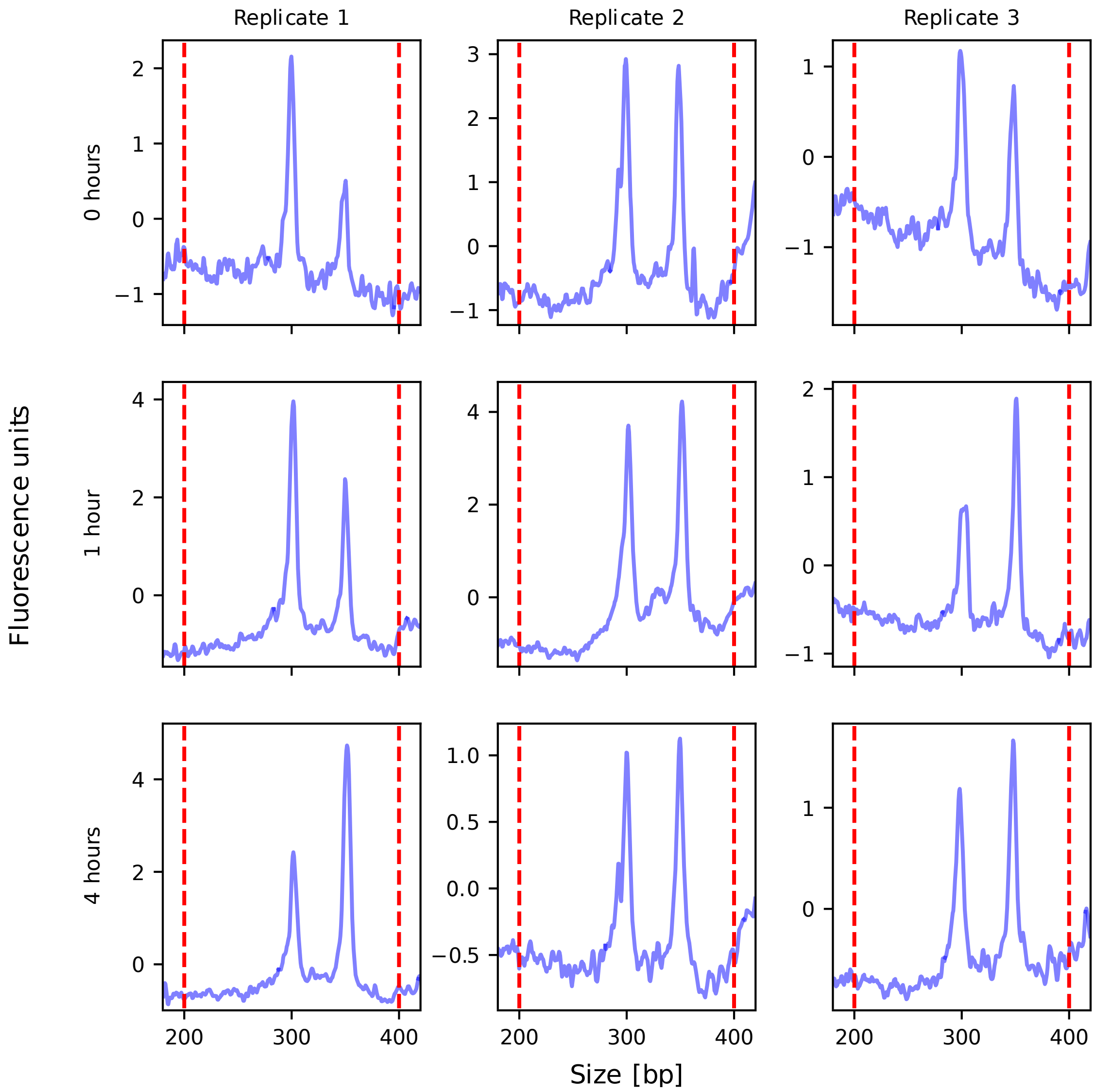

Supplementary Figure S1B. Product sizes of selected paired-end libraries of short capped RNAs before size selection were measured by Bioanalyzer. The product size distribution is shown for the selected size range. The selected size limits are indicated by red dashed lines.

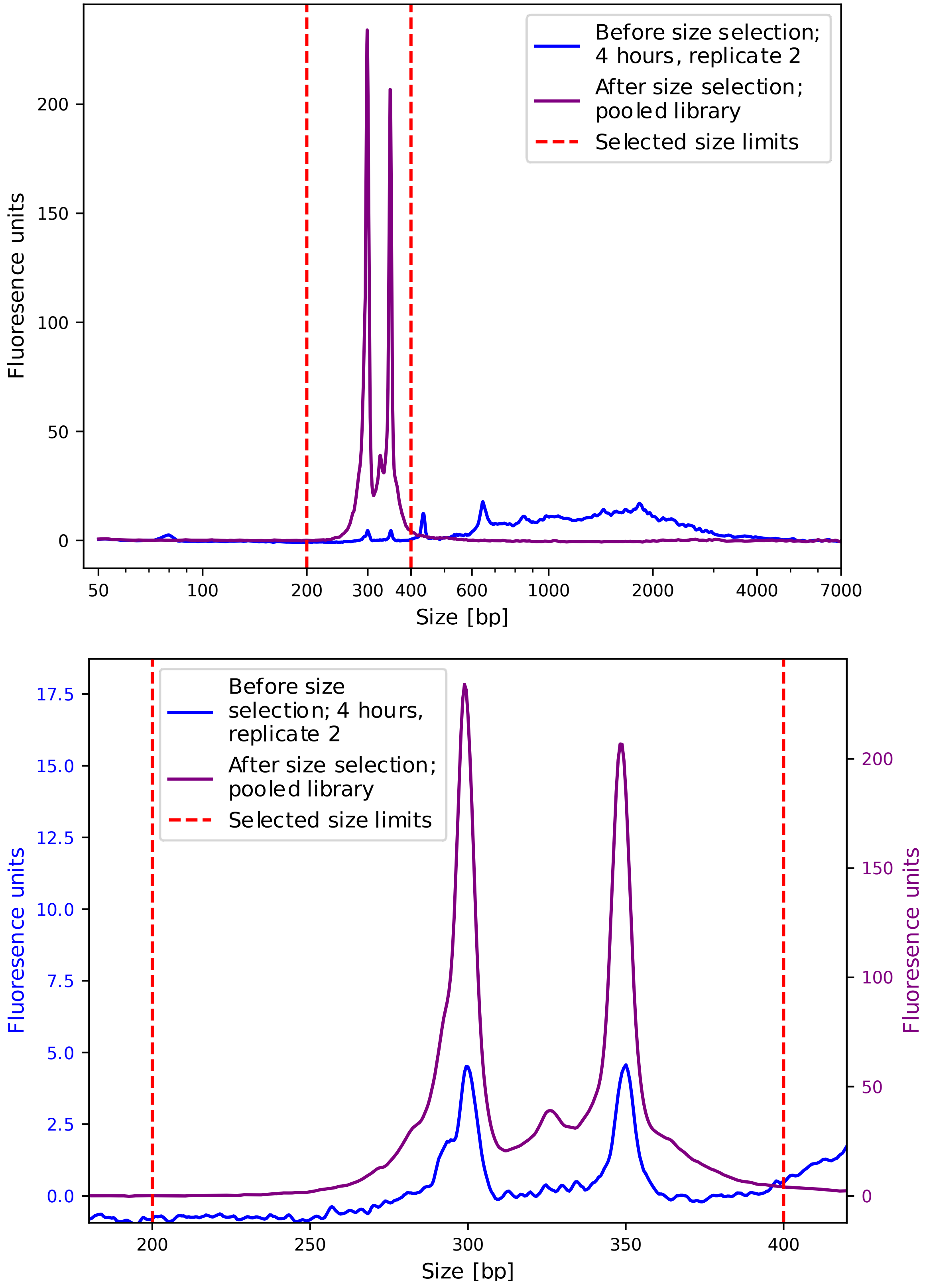

Supplementary Figure S1C. Distribution of product sizes measured by Bioanalyzer in the paired-end libraries of short capped RNAs. The pooled library after size selection and, for comparison, the library for replicate 2 at 4 hours in the time course before pooling and size selection were measured. The selected size limits are indicated by red dashed lines. (top) full size range; (bottom) selected size range.
