# Supplementary material for: Deep sequencing of short capped RNAs reveals novel families of noncoding RNAs
Source: Genome Res. 2022 Sep;32(9):1727–35. doi: 10.1101/gr.276647.122 (PMC9528987; doi:10.1101/gr.276647.122)
Supplement: Supplemental Material [file supp_gr.276647.122_Supplemental_Fig_S2.pdf]

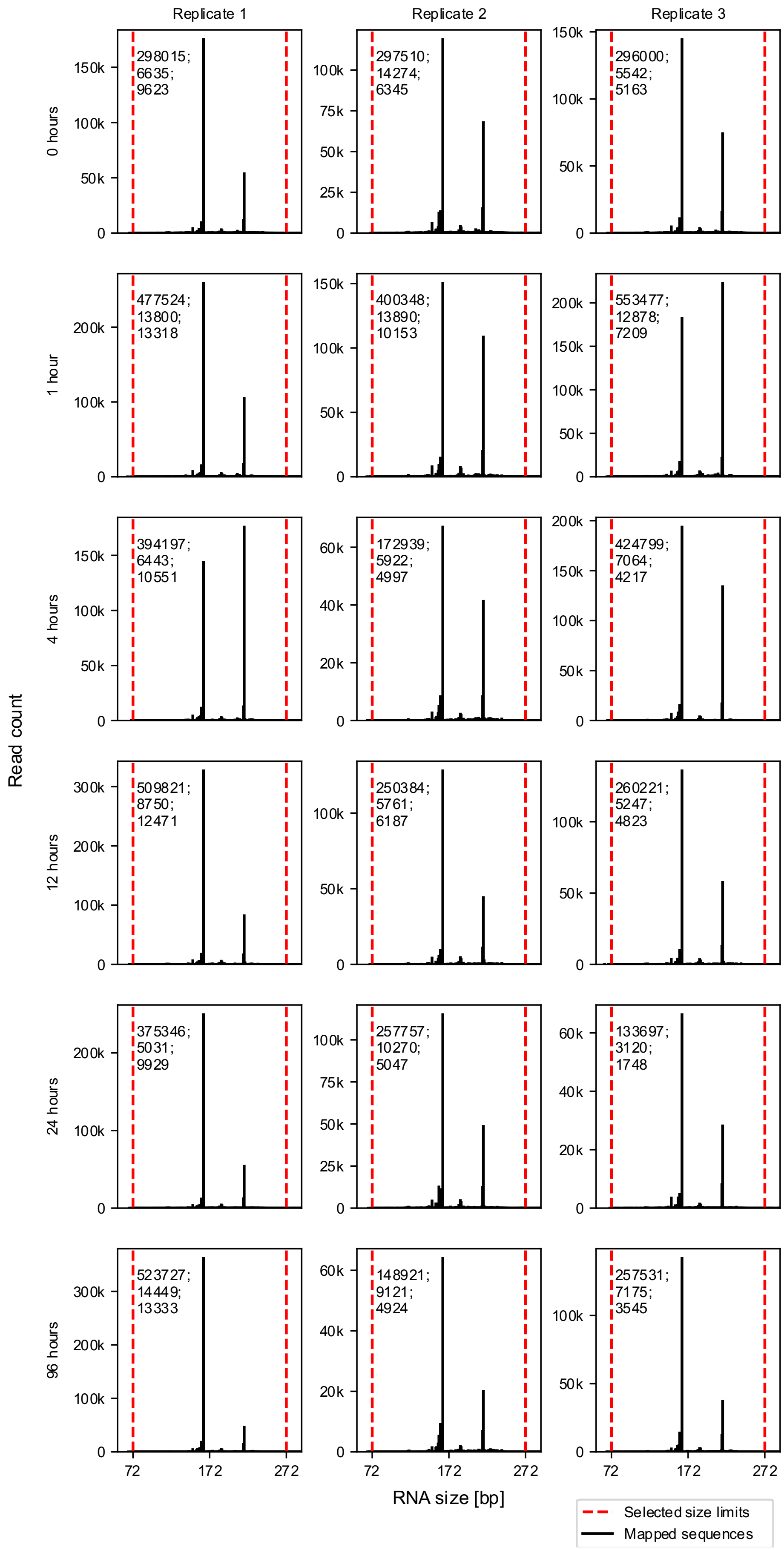
Replicate 1
Replicate 2
Replicate 3
0 hours
1 hour
4 hours
12 hours
24 hours
96 hours
Read count
RNA size [bp]
72
172
272
298015; 6635; 9623
297510; 14274; 6345
296000; 5542; 5163
477524; 13800; 13318
400348; 13890; 10153
553477; 12878; 7209
394197; 6443; 10551
172939; 5922; 4997
424799; 7064; 4217
509821; 8750; 12471
250384; 5761; 6187
260221; 5247; 4823
375346; 5031; 9929
257757; 10270; 5047
133697; 3120; 1748
523727; 14449; 13333
148921; 9121; 4924
257531; 7175; 3545
Selected size limits
Mapped sequences

Supplementary Figure S2.

Distribution of RNA sizes inferred from the paired-end sequencing data of short capped RNAs. The total number of mapped sequences, the number of mapped sequences with inferred RNA sizes outside of the window shown, and the number of unmapped sequences are indicated in each panel.
