# Supplementary material for: Deep sequencing of short capped RNAs reveals novel families of noncoding RNAs
Source: Genome Res. 2022 Sep;32(9):1727–35. doi: 10.1101/gr.276647.122 (PMC9528987; doi:10.1101/gr.276647.122)
Supplement: Supplemental Material [file supp_gr.276647.122_Supplemental_Fig_S3.pdf]

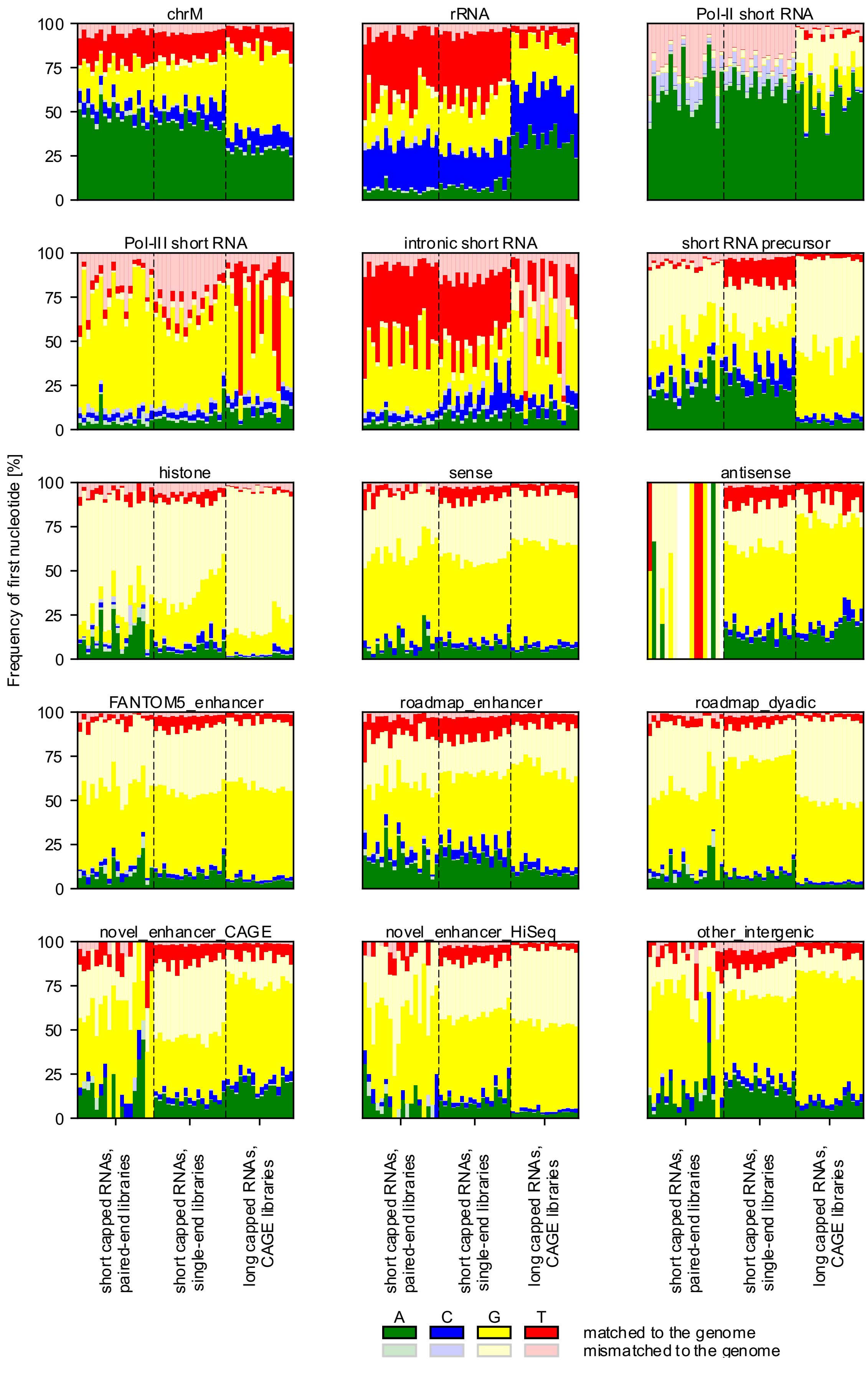
chrM
rRNA
Pol-II short RNA
Pol-III short RNA
intronic short RNA
short RNA precursor
histone
sense
antisense
FANTOM5_enhancer
roadmap_enhancer
roadmap_dyadic
novel_enhancer_CAGE
novel_enhancer_HiSeq
other_intergenic
Frequency of first nucleotide [%]
0
25
50
75
100
short capped RNAs, paired-end libraries
short capped RNAs, single-end libraries
long capped RNAs, CAGE libraries
A
C
G
T
matched to the genome
mismatched to the genome

Supplementary Figure S3.

Enrichment of G nucleotides at the first base of the sequenced read, indicative of transcription by RNA polymerase II, for different classes of transcripts. RNA classes shown in the title of each panel are defined as in Figure 1; samples are shown in the same order as in Figure 1. As the reverse transcriptase tends to add a cytosine to the cDNA when encountering the 5'-linked guanine nucleotide of the cap during reverse transcription of RNAs generated by RNA polymerase II, most sequenced reads of capped RNAs have an additional G at their 5' end. To confirm that the RNAs we observed are indeed independent products of RNA polymerase II, we counted the nucleotide frequency at the first base of the sequenced reads. For each class of short capped transcripts, we found a strong enrichment of G nucleotides. Exceptions were transcripts aligning to the mitochondrial genome, ribosomal RNA, RNAs produced by RNA polymerase III, and short RNAs excised from introns, which are not capped, as well as products of RNA polymerase II for which the cap is known to undergo 2,2,7-trimethylation, which prevents cytosine from base-pairing to the 5'-linked guanine.
