# Supplementary material for: Deep sequencing of short capped RNAs reveals novel families of noncoding RNAs
Source: Genome Res. 2022 Sep;32(9):1727–35. doi: 10.1101/gr.276647.122 (PMC9528987; doi:10.1101/gr.276647.122)
Supplement: Supplemental Material [file supp_gr.276647.122_Supplemental_Fig_S4.pdf]

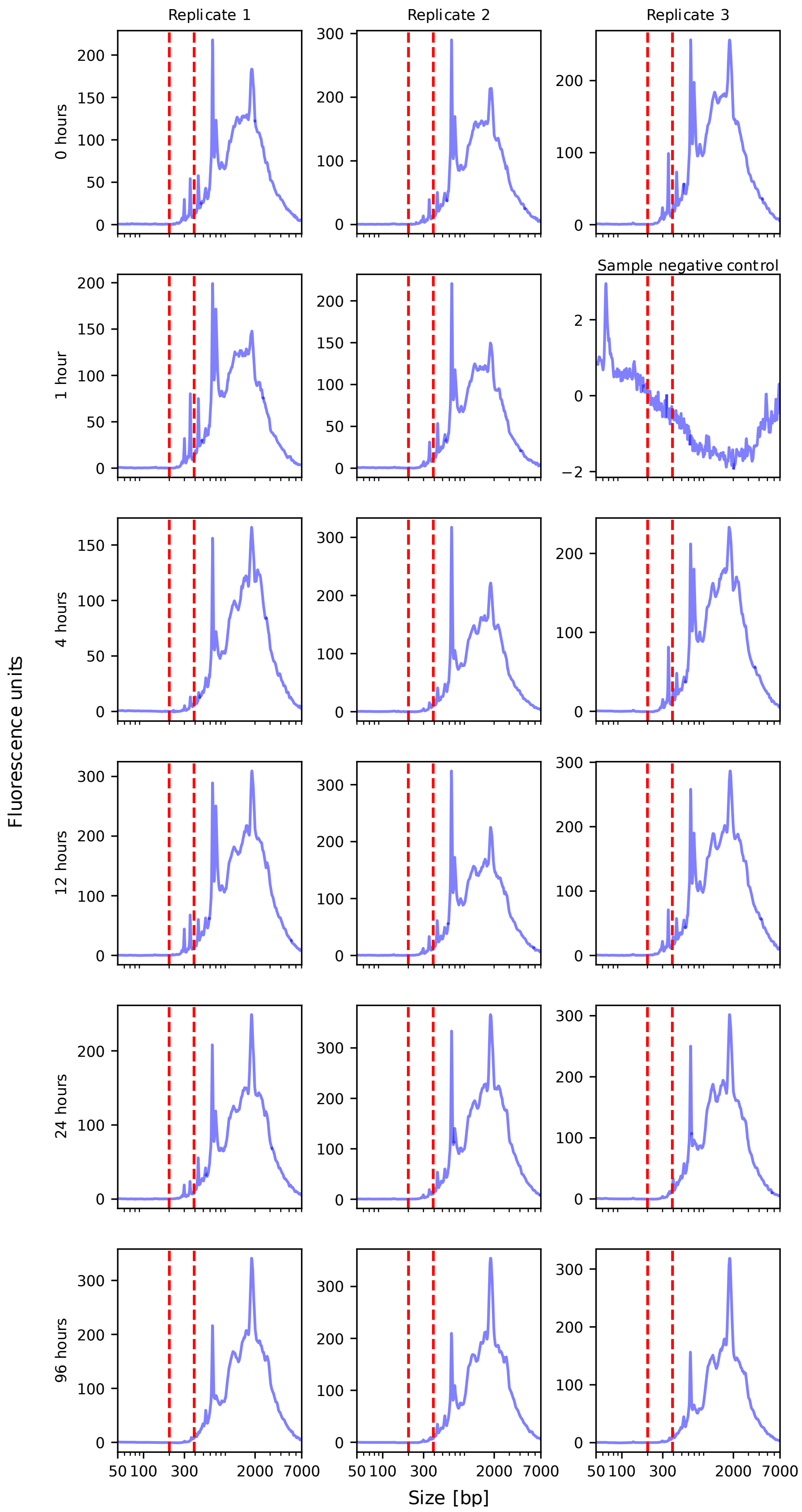

Supplementary Figure S4A. Distribution of product sizes measured by Bioanalyzer in the single-end libraries of short capped RNAs, before size selection; full size range. The selected size limits are indicated by red dashed lines.

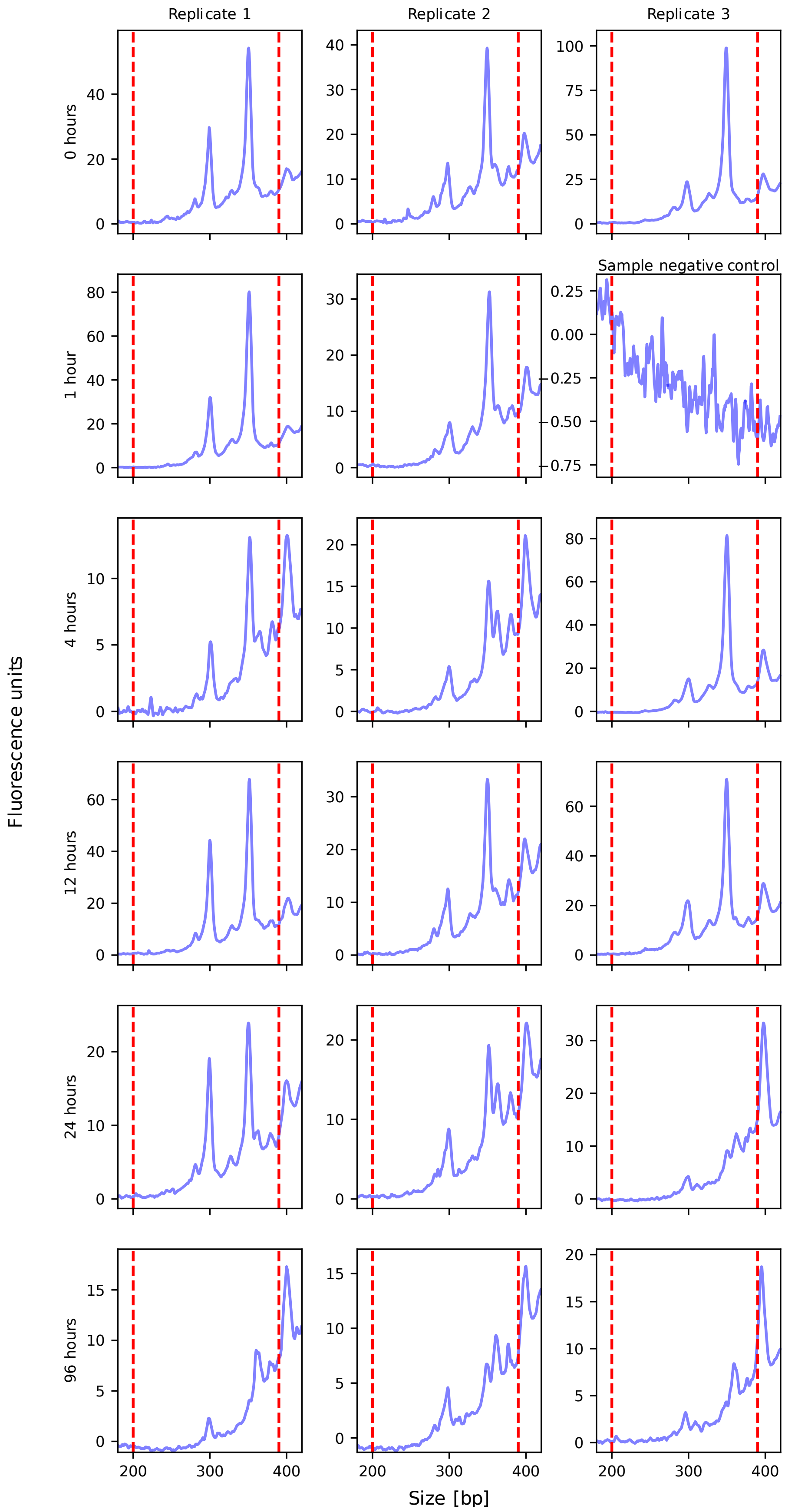

Supplementary Figure S4B. Distribution of product sizes measured by Bioanalyzer in the single-end libraries of short capped RNAs, before size selection; selected size range. The selected size limits are indicated by red dashed lines.

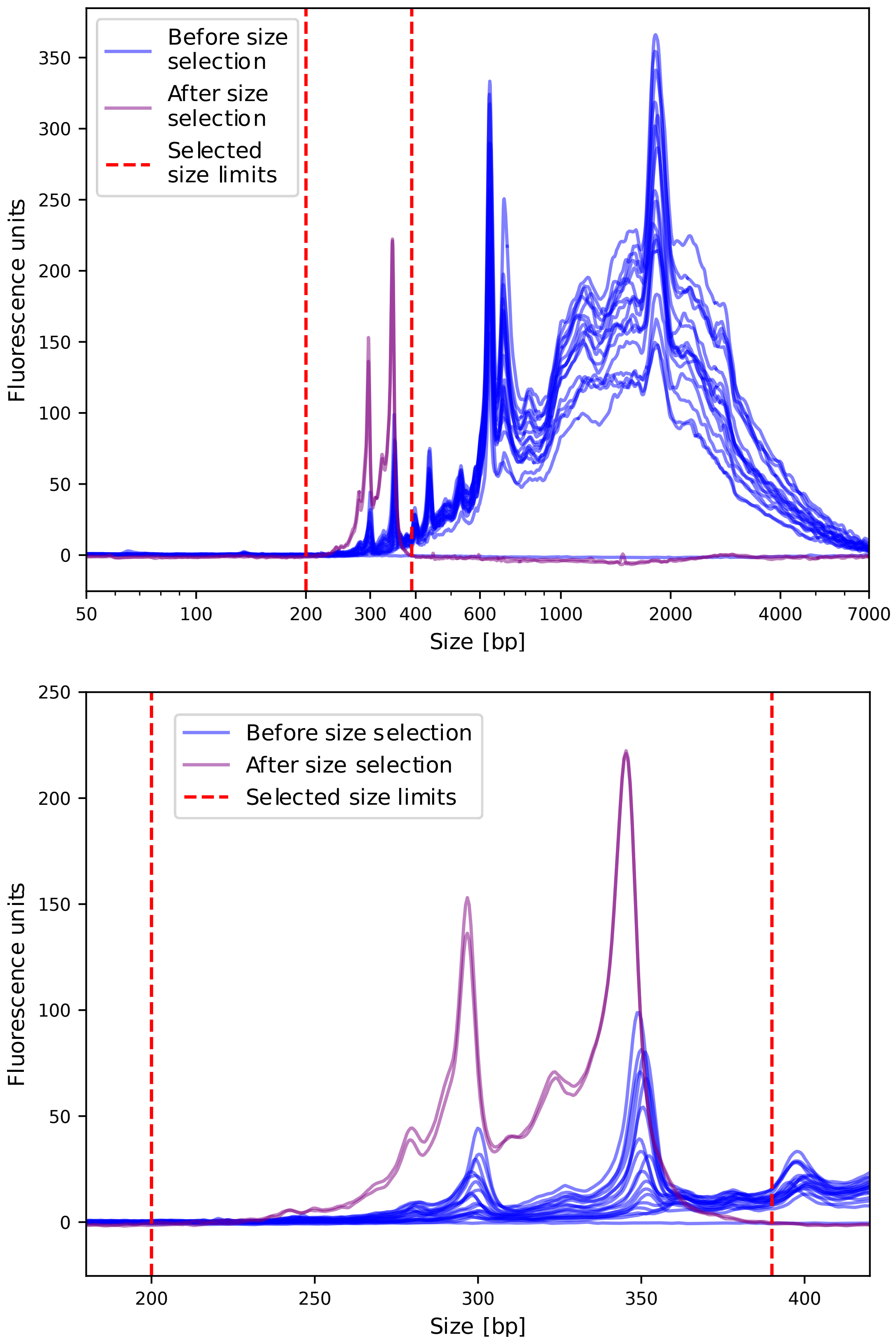

Supplementary Figure S4C. Distribution of product sizes measured by Bioanalyzer after size selection of the pooled 18-mix single-end library of short capped RNAs, together with the distribution for each library separately before pooling and size selection. The selected size limits are indicated by red dashed lines. (top) full size range; (bottom) selected size range.
