# Supplementary material for: Deep sequencing of short capped RNAs reveals novel families of noncoding RNAs
Source: Genome Res. 2022 Sep;32(9):1727–35. doi: 10.1101/gr.276647.122 (PMC9528987; doi:10.1101/gr.276647.122)
Supplement: Supplemental Material [file supp_gr.276647.122_Supplemental_Fig_S5.pdf]

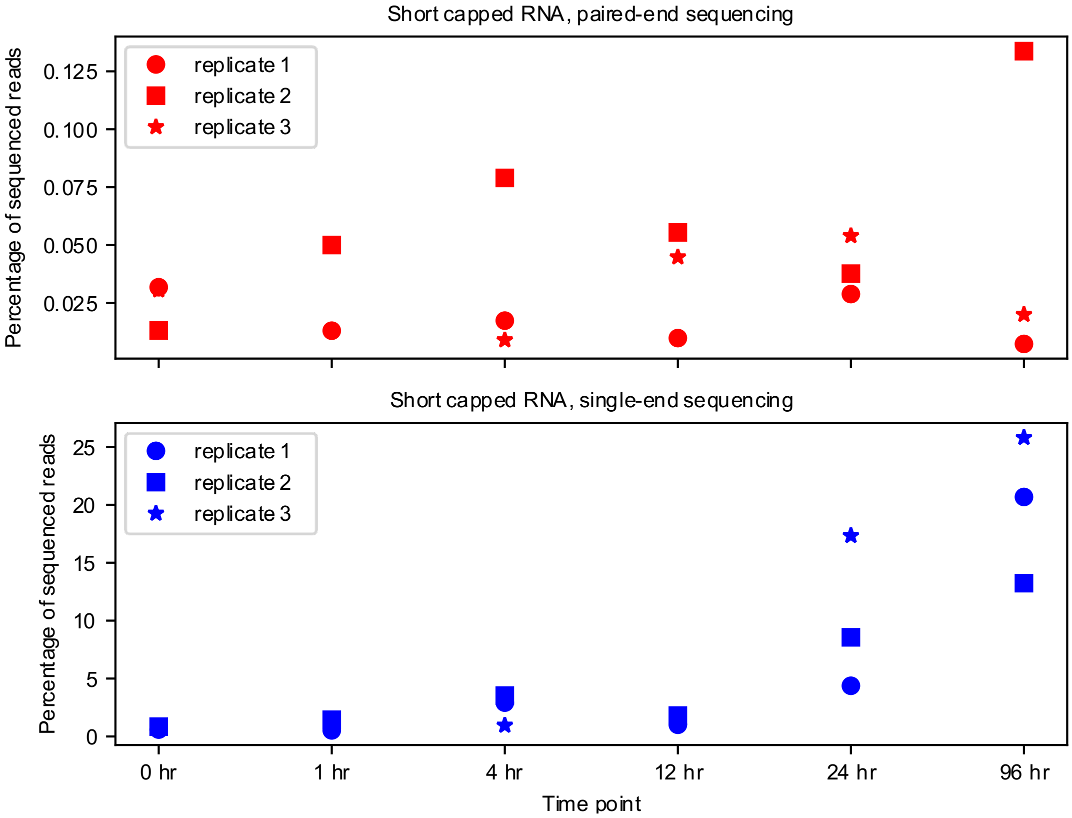

Supplementary Figure S5A. Percentage of sequenced reads aligning to the DBI promoter region in the paired-end (top) and single-end (bottom) libraries of short capped RNAs.

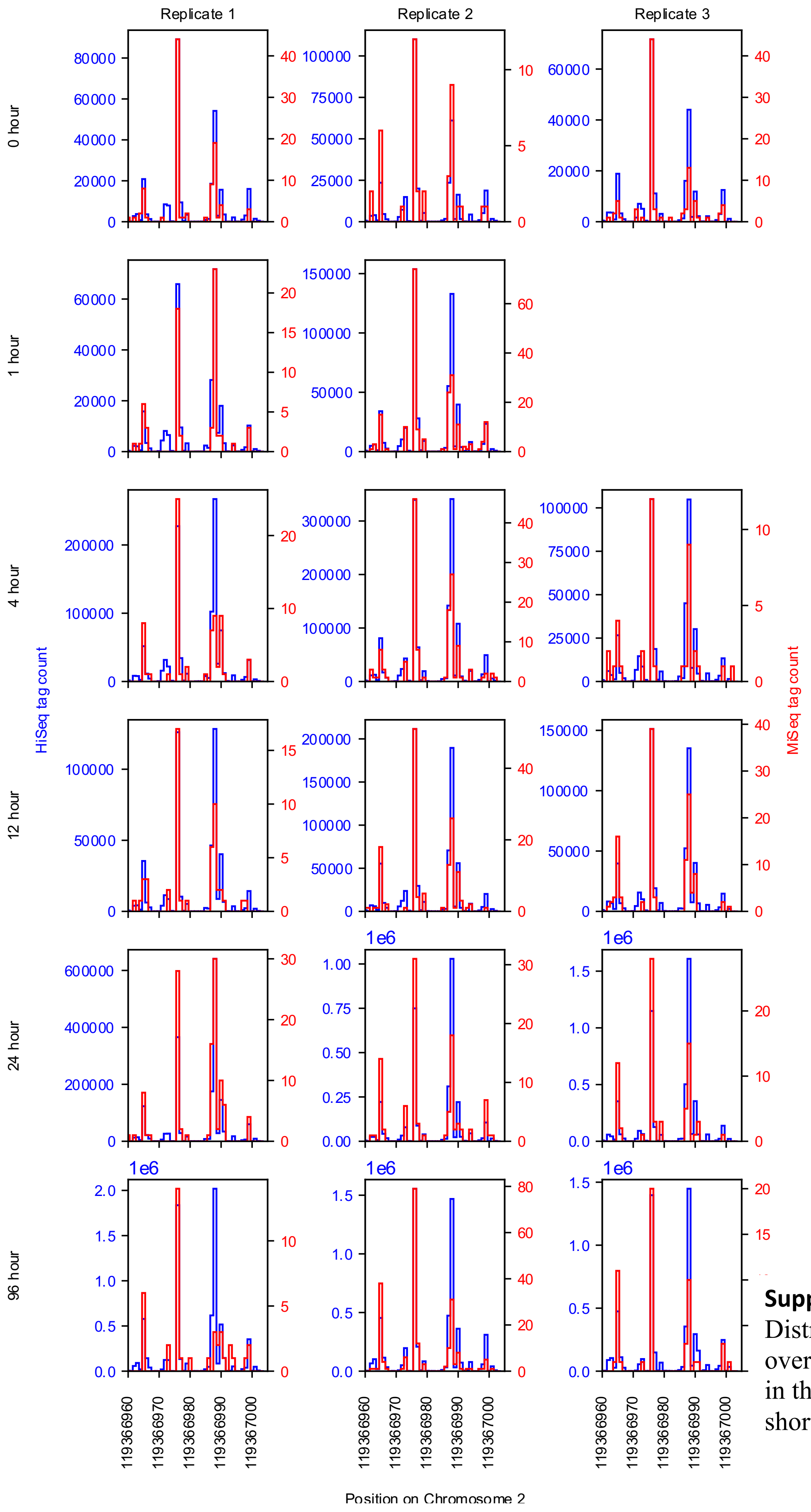

Supplementary Figure S5B. Distribution of sequenced reads over the DBI promoter region in the single-end libraries of short capped RNAs.

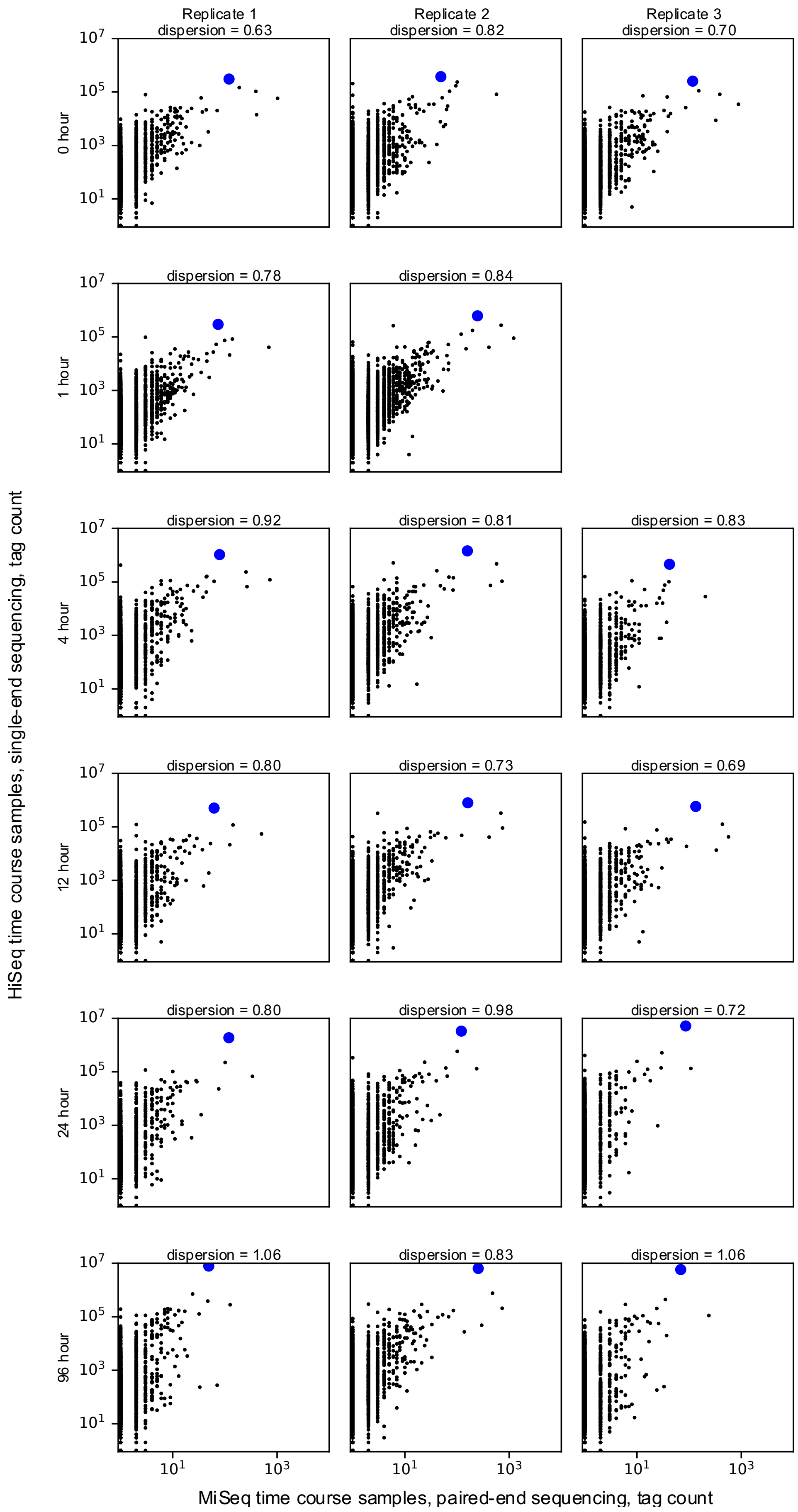

Supplementary Figure S5C. Scatter plots comparing the expression level of short capped RNAs in the THP-1 time course between the paired-end and the single-end libraries of short capped RNAs. Each dot represents one transcription initiation peak on the genome. The peak at the DBI promoter is indicated in blue. The global dispersion value is shown in the title of each panel.

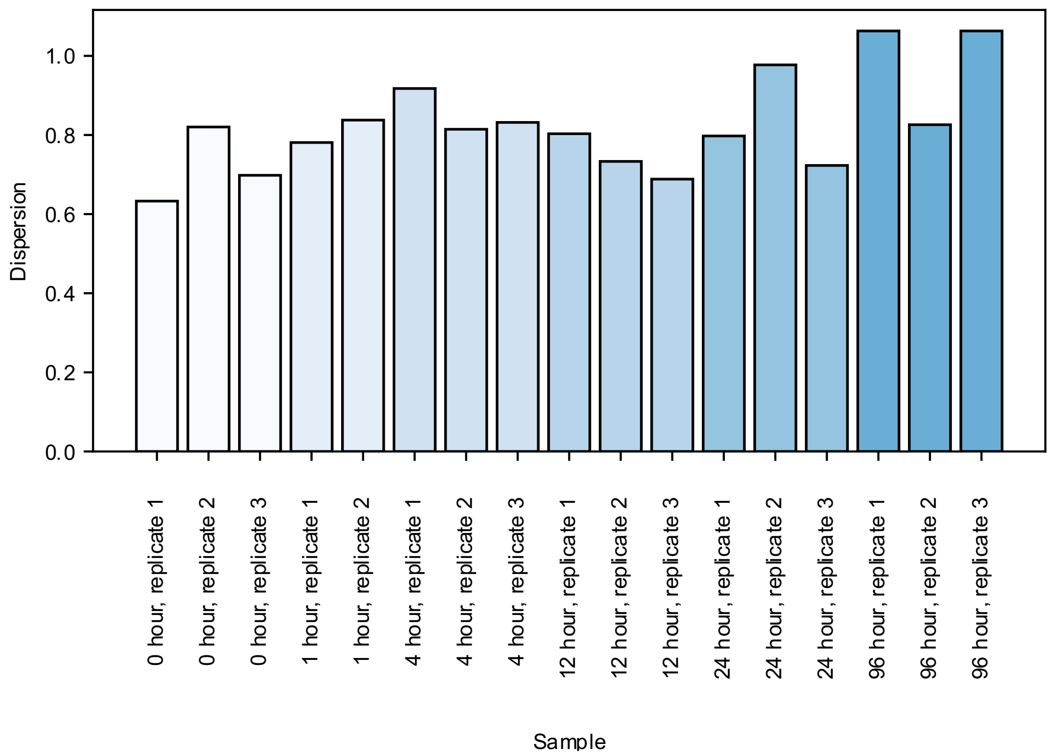

Supplementary Figure S5D.
Global dispersion value between the paired-end and single-end short capped RNA library for each time point and replicate.
