# Supplementary material for: Deep sequencing of short capped RNAs reveals novel families of noncoding RNAs
Source: Genome Res. 2022 Sep;32(9):1727–35. doi: 10.1101/gr.276647.122 (PMC9528987; doi:10.1101/gr.276647.122)
Supplement: Supplemental Material [file supp_gr.276647.122_Supplemental_Fig_S7.pdf]

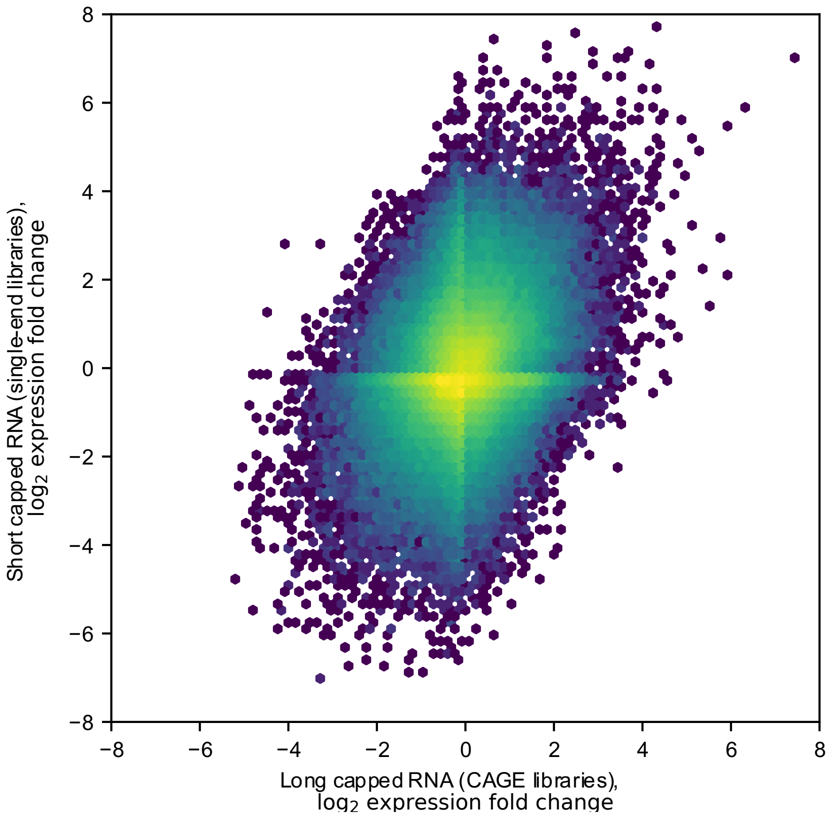

Supplementary Figure S7. Differential expression of long capped RNAs (as observed in the CAGE libraries) during the time course, and differential expression of short capped RNAs (as observed in the single-end libraries) originating from the same gene-associated transcription initiation peak. Log_2 expression fold changes were calculated using the variance stabilizing transformation (see Methods).
