# Supplementary material for: Deep sequencing of short capped RNAs reveals novel families of noncoding RNAs
Source: Genome Res. 2022 Sep;32(9):1727–35. doi: 10.1101/gr.276647.122 (PMC9528987; doi:10.1101/gr.276647.122)
Supplement: Supplemental Material [file supp_gr.276647.122_Supplemental_Fig_S8.pdf]

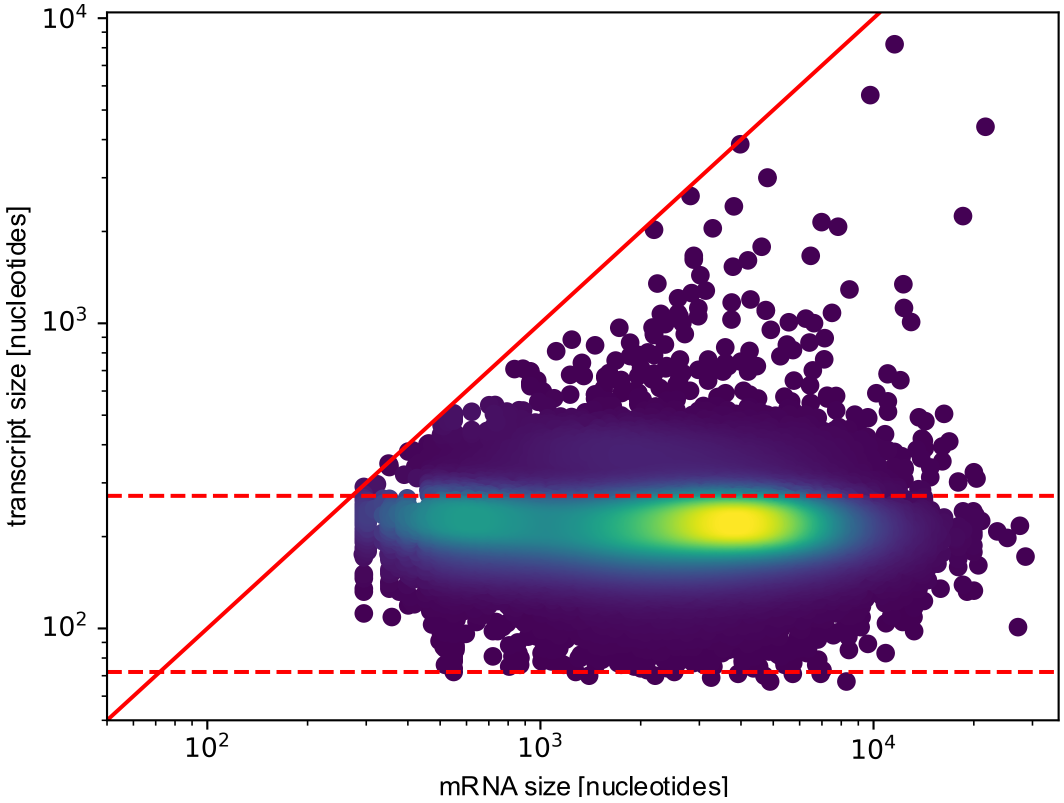

Supplementary Figure S8A. Transcript size of short capped RNAs aligning to mature mRNA transcripts versus the size of the mRNA transcript, colored by local density, using the paired-end sequencing data. The peak consists of transcripts aligning to mature lncRNA transcripts, but with a premature 3’ end (see **Figure 3A**). Dashed red lines indicate the transcript sizes that were selected by library size selection. The solid red line is the identity line.

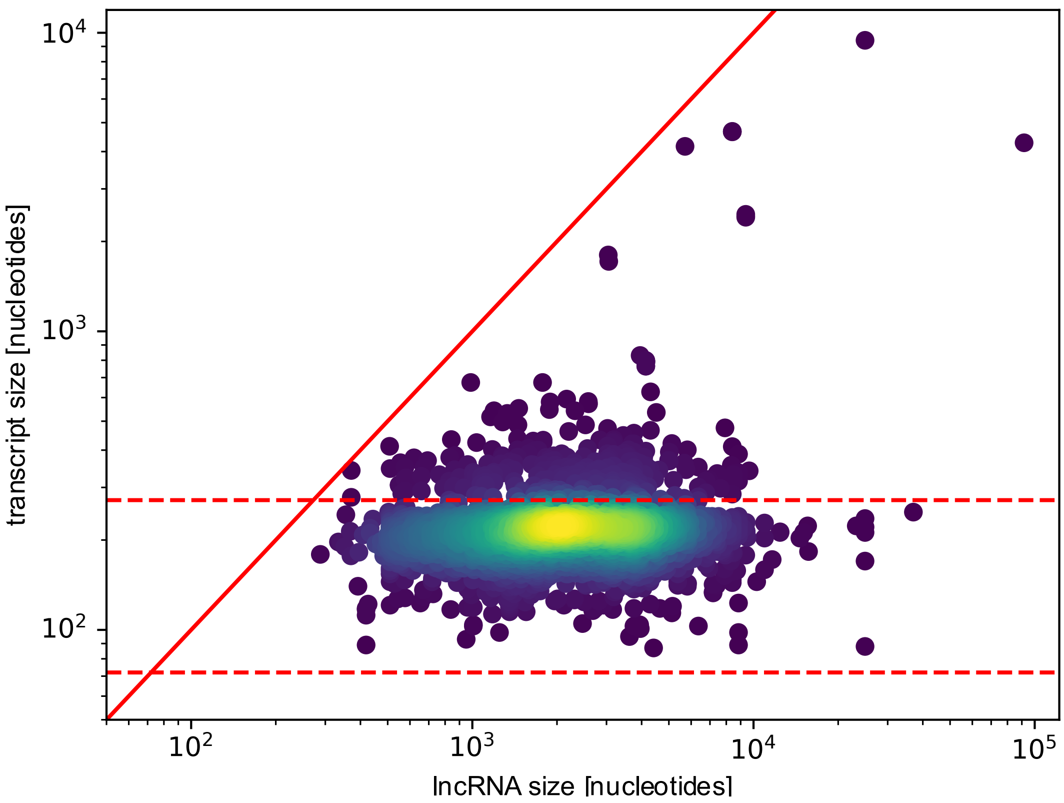

Supplementary Figure S8B. Transcript size of short capped RNAs aligning to mature lncRNA transcripts versus the size of the lncRNA transcript, colored by local density, using the paired-end sequencing data. The peak consists of transcripts aligning to mature lncRNA transcripts, but with a premature 3' end (see **Figure 3B**). Dashed red lines indicate the transcript sizes that were selected by cDNA size selection. The solid red line is the identity line.
