# Supplementary material for: Deep sequencing of short capped RNAs reveals novel families of noncoding RNAs
Source: Genome Res. 2022 Sep;32(9):1727–35. doi: 10.1101/gr.276647.122 (PMC9528987; doi:10.1101/gr.276647.122)
Supplement: Supplemental Material [file supp_gr.276647.122_Supplemental_Fig_S9.pdf]

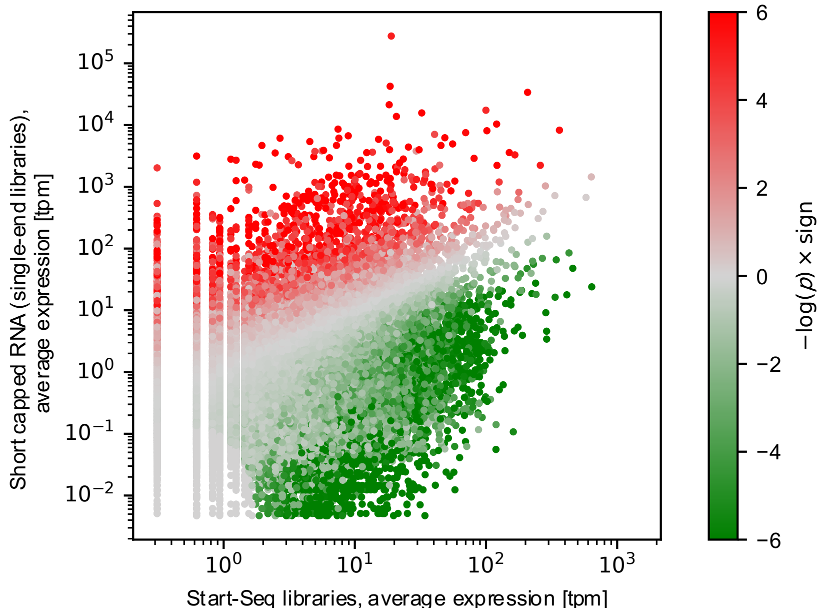

Supplementary Figure S9A. Scatter plot of the expression observed in the Start-Seq libraries and in the single-end libraries (short capped RNAs) of gene-associated transcription initiation peaks. The expression was normalized to tags per million (tpm), averaged over replicates at each time point, and for the short capped RNA libraries averaged over the time points. Colors represent the base-10 logarithm of the adjusted *p*-value of differential expression as reported by DESeq2.

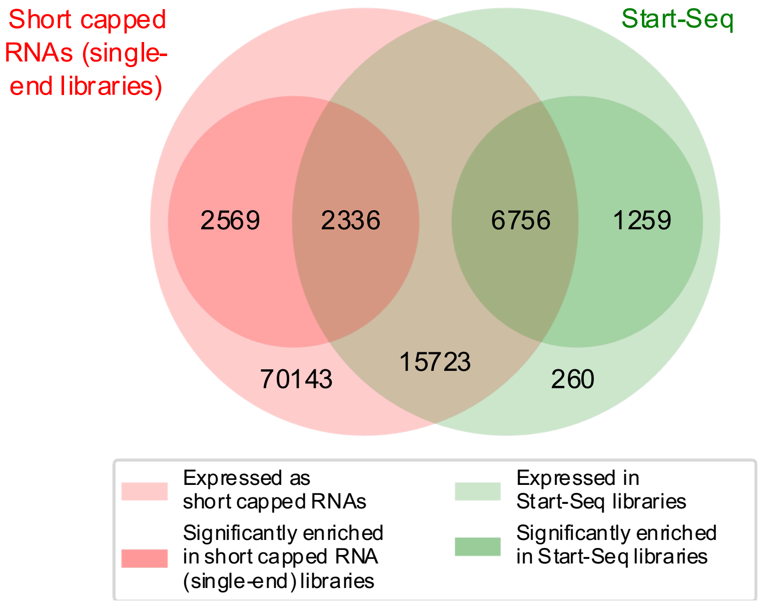

Supplementary Figure S9B. Venn diagram of transcription initiation peaks associated with genes in the sense orientation. The outer circles represent peaks expressed in the single-end libraries of short capped RNAs (red outer circle) and in the Start-Seq libraries (green outer circle). The inner circles represent peaks significantly enriched in the single-end libraries of short capped RNAs compared to the Start-Seq libraries (red inner circle), or a significantly enriched in the Start-Seq libraries compared to the single-end libraries of short capped RNAs (green inner circle).
