# Supplementary material for: Deep sequencing of short capped RNAs reveals novel families of noncoding RNAs
Source: Genome Res. 2022 Sep;32(9):1727–35. doi: 10.1101/gr.276647.122 (PMC9528987; doi:10.1101/gr.276647.122)
Supplement: Supplemental Material [file supp_gr.276647.122_Supplemental_Fig_S10.pdf]

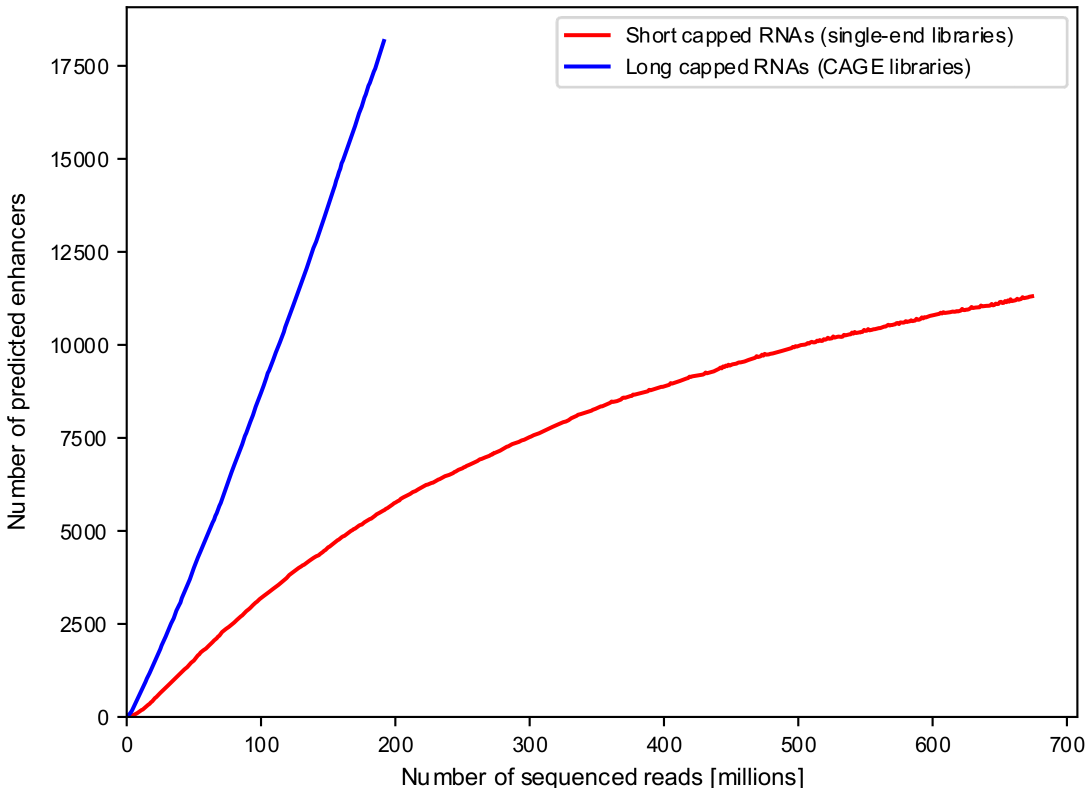

Supplementary Figure S10. Number of predicted enhancers as a function of sequencing depth. Both for short capped RNAs (single-end libraries) and long capped RNAs (CAGE libraries), enhancer prediction has not reached saturation.
