# Supplementary material for: Deep sequencing of short capped RNAs reveals novel families of noncoding RNAs
Source: Genome Res. 2022 Sep;32(9):1727–35. doi: 10.1101/gr.276647.122 (PMC9528987; doi:10.1101/gr.276647.122)
Supplement: Supplemental Material [file supp_gr.276647.122_Supplemental_Fig_S11.pdf]

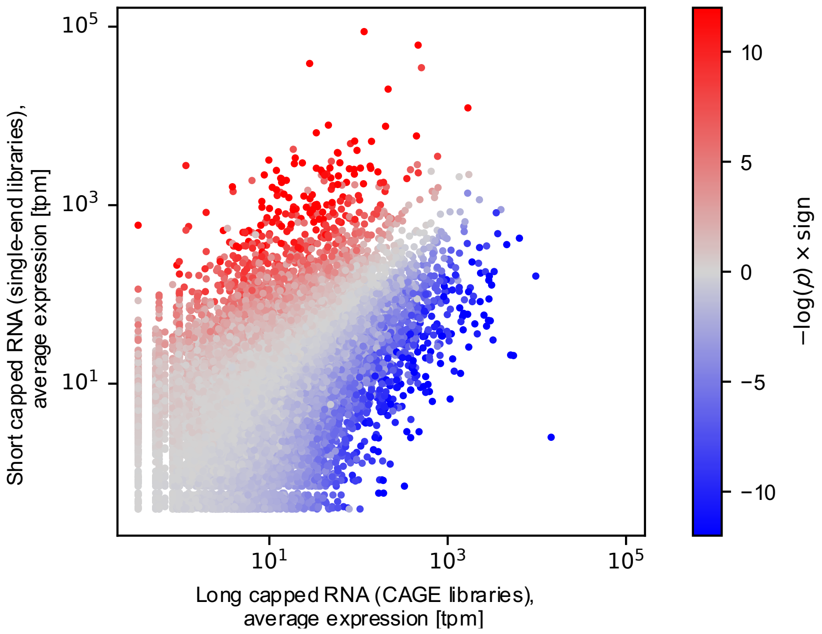

Supplementary Figure S11. Scatter plot of the expression of long capped RNAs (in the CAGE libraries) and short capped RNAs (in the single-end libraries) originating from the same enhancer. The expression was normalized to tags per million (tpm) relative to the total number of tags associated with enhancers, averaged over replicates at each time point, and then averaged over the six time points in the time course. Colors represent the base-10 logarithm of the adjusted *p*-value of differential expression as reported by DESeq2.
